# Supplementary material for: A bespoke mobile application for the longitudinal assessment of depression and mood during pregnancy: protocol of a feasibility study
Source: BMJ Open. 2017 May 29;7(5):e014469. doi: 10.1136/bmjopen-2016-014469 (PMC5729976; doi:10.1136/bmjopen-2016-014469)
Supplement: Supplementary appendix 1 [file bmjopen-2016-014469supp001.pdf]

Appendix 1. Baseline assessments

Socio-demographic survey

- What is your age group?
  - 18 to 22 years
  - 23 to 27 years
  - 28 to 32 years
  - 33 to 37 years
  - 38 years or older
- How would you describe your race or ethnicity?
  - White
    - British
    - Irish
    - Other
  - Mixed
    - White and Black Caribbean
    - White and Black African
    - White and Asian
    - Other
  - Asian or Asian British
    - Indian
    - Pakistani
    - Bangladeshi
    - Other
  - Black or Black British
    - Caribbean
    - African
    - Other
  - Chinese
  - Other
  - Prefer not to say
- What is your relationship status?
  - Single
  - Married/In a civil partnership
  - Living with partner
  - Divorced/Civil partnership that has been dissolved
  - Widowed
  - Separated
  - Prefer not to say
- What is your employment status?
  - Employed, full-time
  - Employed, part-time
  - Self-employed
  - Not employed, looking for work
  - Not employed, not looking for work
  - Disability/Not able to work

- What is your highest level of education?
  - Postgraduate – PhD/Doctorate
  - Postgraduate – Masters degree
  - University or college degree
  - University or college qualification below degree level
  - A Levels or equivalent
  - GCSE or equivalent
  - Other
  - None of these
- Do you own a smartphone (for example, an iPhone or Samsung phone)?
  - Yes
  - No
- Do you own a tablet computer (for example, an iPad)?
  - Yes
  - No
- How many times have you been pregnant (please count your current pregnancy)?
  - 1
  - 2
  - 3
  - 4 or more
- How many children have you given birth to?
  - 0
  - 1
  - 2
  - 3 or more
- When is your baby due? (please indicate month and year)
- Have you ever been diagnosed with depression?
  - Yes
  - No

Whooley questions

- Over the past month, have you been bothered by feeling down, depressed or hopeless?
  - Yes
  - No
- Over the past month, have you been bothered by having little interest or pleasure in doing things?
  - Yes
  - No

Edinburgh Postnatal Depression Scale

As you are pregnant or have recently had a baby, we would like to know how you are feeling. Please check the answer that comes closest to how you have felt IN THE PAST 7 DAYS, not just how you feel today.

In the past 7 days:

1. I have been able to laugh and see the funny side of things
    a. As much as I always could
    b. Not quite so much now
    c. Definitely not so much now
    d. Not at all
2. I have looked forward with enjoyment to things
    a. As much as I ever did
    b. Rather less than I used to
    c. Definitely less than I used to
    d. Hardly at all
3. I have blamed myself unnecessarily when things went wrong
    a. Yes, most of the time
    b. Yes, some of the time
    c. Not very often
    d. No, never
4. I have been anxious or worried for no good reason
    a. No, not at all
    b. Hardly ever
    c. Yes, sometimes
    d. Yes, very often
5. I have felt scared or panicky for no very good reason
    a. Yes, quite a lot
    b. Yes, sometimes
    c. No, not much
    d. No, not at all
6. Things have been getting on top of me
    a. Yes, most of the time I haven't been able to cope at all
    b. Yes, sometimes I haven't been coping as well as usual
    c. No, most of the time I have coped quite well
    d. No, I have been coping as well as ever
7. I have been so unhappy that I have had difficulty sleeping
    a. Yes, most of the time
    b. Yes, sometimes
    c. Not very often
    d. No, not at all
8. I have felt sad or miserable
    a. Yes, most of the time
    b. Yes, quite often
    c. Not very often
    d. No, not at all
9. I have been so unhappy that I have been crying
    a. Yes, most of the time
    b. Yes, quite often
    c. Only occasionally
    d. No, never
10. The thought of harming myself has occurred to me
    a. Yes, quite often
    b. Sometimes
    c. Hardly ever
    d. Never
