# Supplementary material for: A bespoke mobile application for the longitudinal assessment of depression and mood during pregnancy: protocol of a feasibility study
Source: BMJ Open. 2017 May 29;7(5):e014469. doi: 10.1136/bmjopen-2016-014469 (PMC5729976; doi:10.1136/bmjopen-2016-014469)
Supplement: Supplementary appendix 2 [file bmjopen-2016-014469supp002.pdf]

BRIGHTSELF

Welcome Guide (Arm 2)

Version1.0 - 6 Jul 2016

Welcome

Hello, and thank you for collaborating with this study to test the feasibility of BrightSelf, an application for the assessment of psychological wellbeing during pregnancy.

This application and study are the result of a collaboration between Imperial College London and Trinity College Dublin.

Here you will find a quick explanation of BrightSelf, the value it provides to participants, and guidelines for recruitment.

The following section answers any questions the user may have and might be of use to you during recruitment.

Questions Answered

What is BrightSelf?

BrightSelf is a new mobile app designed to help women develop a picture of their psychological wellbeing during pregnancy.

Researchers at Imperial College London and Trinity College Dublin are currently assessing the engagement of mothers-to-be with BrightSelf. In future, we hope to assess the value of the app to improve the wellbeing of mothers-to-be and communication between women and their healthcare providers.

By **checking in** you can capture your mood, sleep, energy and more in the moment. By **checking back**, BrightSelf allows you to build a picture of your wellbeing over the past week. BrightSelf also provides the ability to interact with your data, information on wellbeing and research, sources of professional support, and an ideas machine, your personal source for a pick-me-up!

What is expected of users?

BrightSelf is here for you to use in any way you find useful. You can make self reports according to a schedule you determine for yourself or simply as often as you feel useful. We have tried to design BrightSelf so that it is quick and easy to use and ultimately supports your needs.

As part of our research to examine the value of BrightSelf and technologies for the report of psychological wellbeing, you will be prompted infrequently over the course of 6 months to check and and check back using the application.

These prompts will appear as notifications on the front screen of your phone or as a banner appearing at the top.

These reports will be used entirely anonymously to inform our research and we would greatly appreciate if you could respond to the requests as accurately and timely as possible. At the end of this period you may then also be asked to complete a short questionnaire detailing your thoughts on the experience.

Through your feedback, and by examining, anonymously and securely, these responses, we hope to move towards improving the experience of all around the time of pregnancy.

The next section provides a quick guide to the installation and use of BrightSelf.

A Quick Guide

The Android Play Store Icon

The Apple App Store Icon

To use BrightSelf, the first step is to download the app to a phone by visiting the appropriate **app store**.

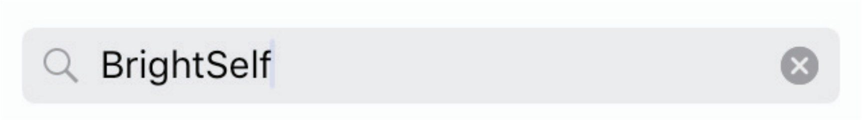

You can then search the store for BrightSelf. Look for the app icon and when you find it, choose to **install** it. It's free!

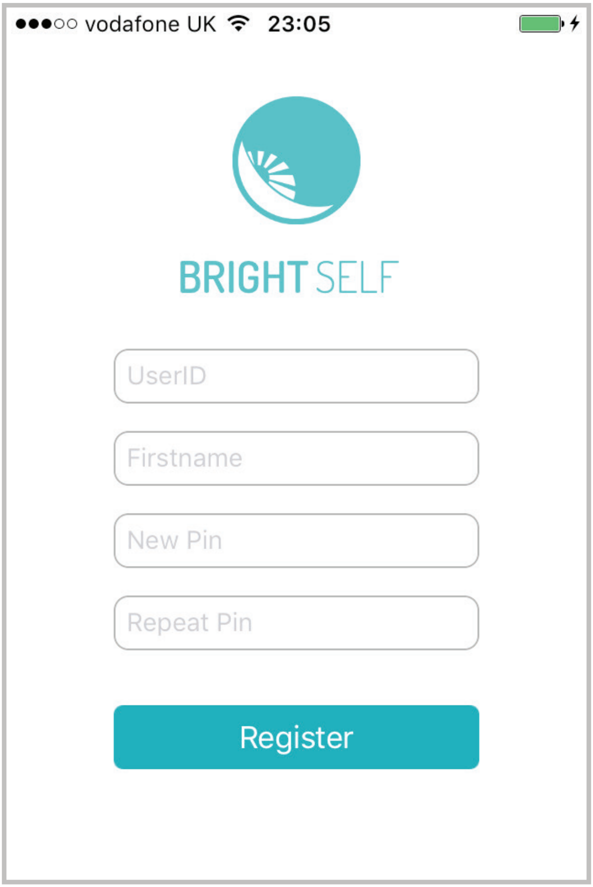

When you first open BrightSelf you will need to **register** a new user.

You may first be asked whether you wish to receive **push notifications** from BrightSelf.

If asked, please accept to do so!

In order to register, an internet connection (Wifi or mobile internet) is required. Once the device is connected, the user simply needs to enter the following information;

- **A 9-digit UserID** provided by the researchers. Please enter this carefully.
- The user's **Firstname** only (without numbers or spaces)
- **A 4-digit PIN** of the user's choosing which will allow her to login in the future. Please ensure to pick a pin which will not be forgotten!
- Enter this **PIN** a second time to ensure a match.

On clicking register, the system will then register the user for the study in several seconds. That's it, you're ready to explore BrightSelf!

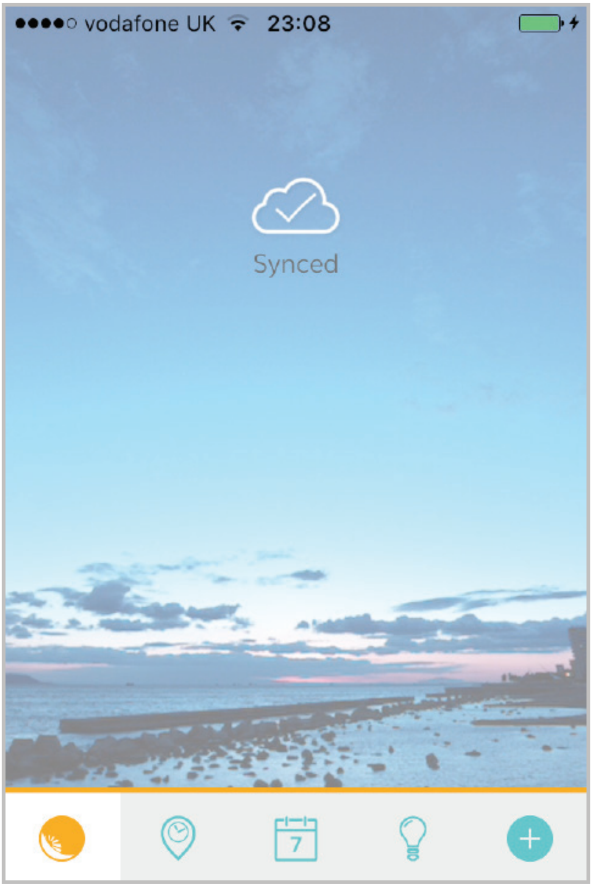

On the **home screen**, the syncing button shows whether your most recent reports have been uploaded, anonymously and securely, to our server for inclusion in our research. You can navigate through the app using the navigation bar located at the bottom of the screen.

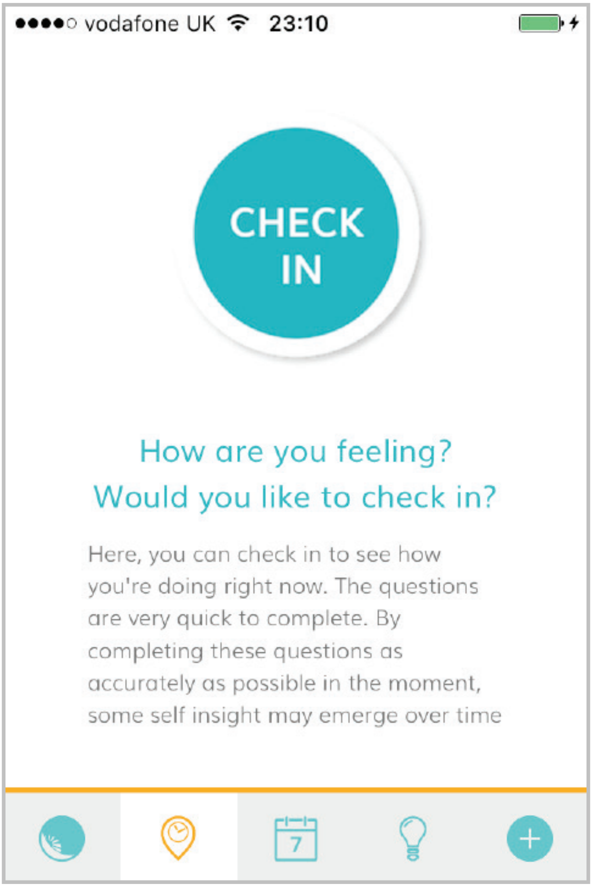

Checking in allows you to note how you are feeling right now by very quickly rating your mood, sleep, worry, enjoyment and energy.

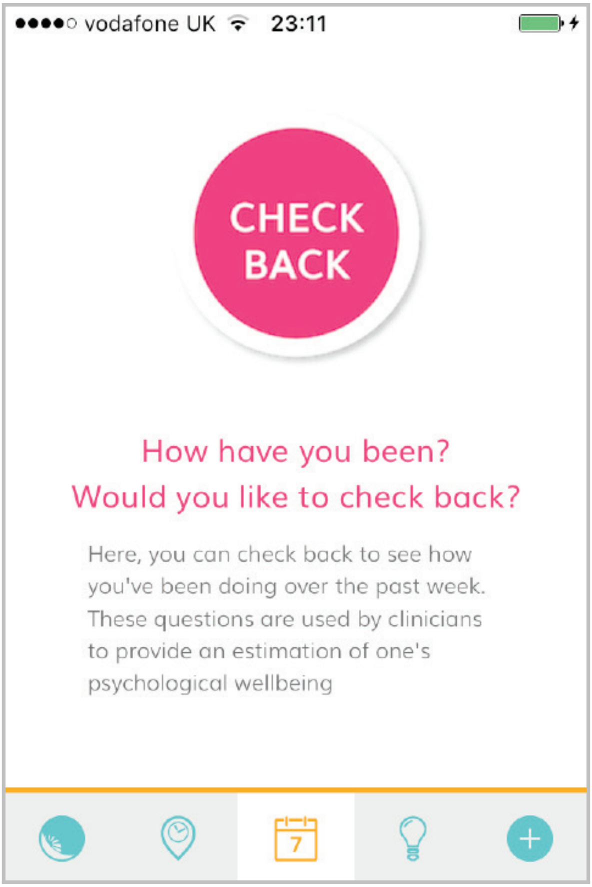

Checking back allows you to make note of your wellbeing over the past week by answering 10 questions which are normally used by clinicians to assess mood during pregnancy.

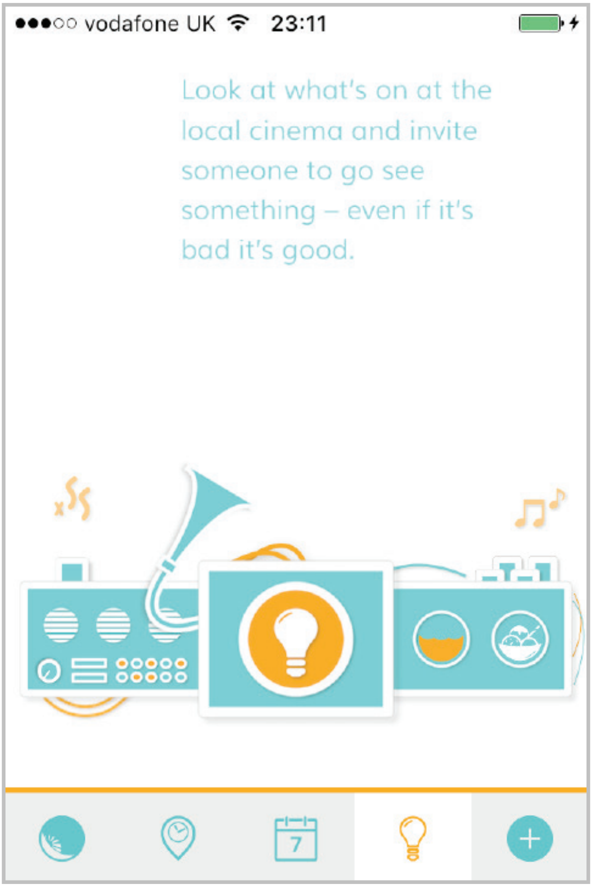

The **ideas machine** is there to provide that pick-me-up we all need from time to time. Remember however, that BrightSelf is not intended as a replacement for professional help. If at any point you feel that you need extra support, please do contact your GP or midwife.

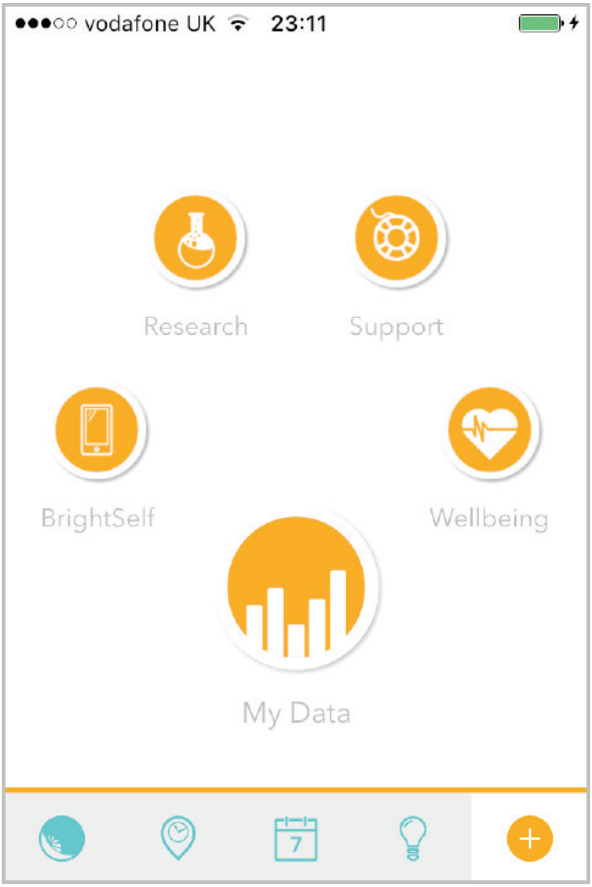

The **extras** section contains information about BrightSelf, wellbeing, and our research. Here you will also find additional sources of professional support should you need them.

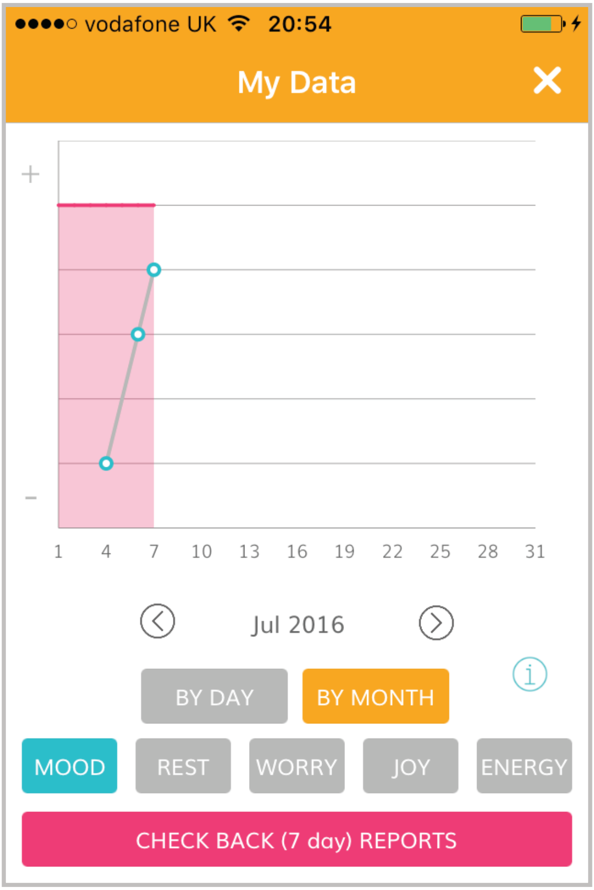

Under **My Data** you can interact with a plot of all your previous responses, allowing you to examine your reports over time, and empowering you to take control of your own wellbeing. Please note however, that these graphs do not represent any proven clinical value.

Finally, please note that you will receive infrequent **notifications** over the next 6 months which will request you to check in and check back. How these notifications appear will depend upon your device and some examples are shown below.

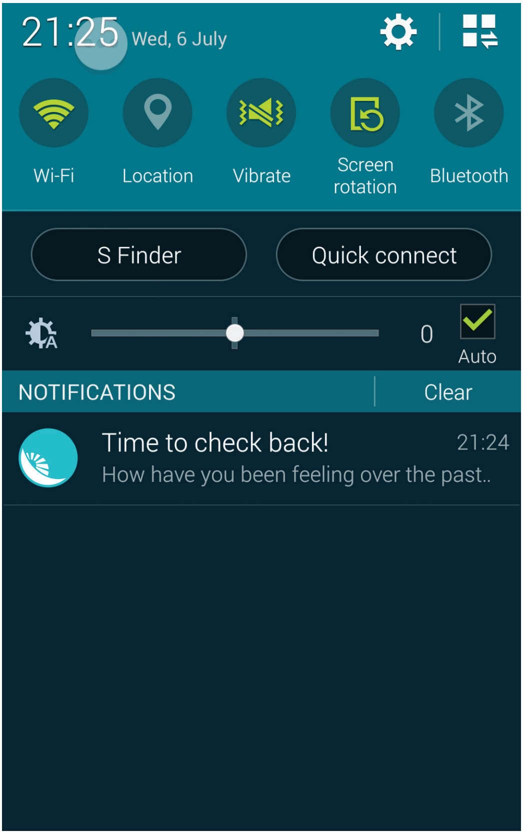

Android Notification

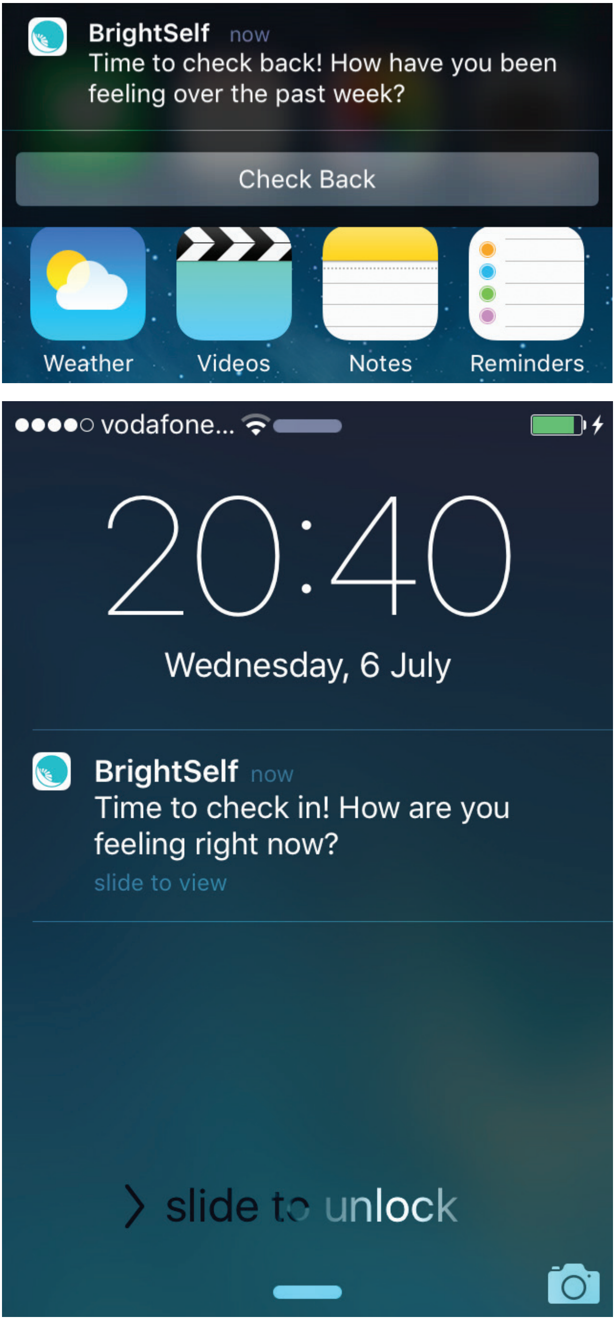

Apple Notification

Administrative Notes

Please note that the user’s data is captured anonymously throughout the duration of the study and transferred to our servers using a secure connection.

No-one outside the research team will have access to the user’s data. Any data gathered will be used for research purposes only.

This app is not intended to substitute the woman’s care in any way but only to complement it. Please inform the user that if at any point she feels the need for extra support to contact her GP or midwife.

If any user’s responses do indicate that she is struggling and might benefit from extra support, the system will alert the research team who will then contact the local research team to follow up.

Contact

At all times, our primary concern is with the user's wellbeing, privacy, and security.

Should you or any user have questions at any time please don't hesitate to contact us at **help@brightselfresearch.org**

Thank you once again for your participation and we hope you find participating in BrightSelf a valuable experience!
