# Supplementary material for: A bespoke mobile application for the longitudinal assessment of depression and mood during pregnancy: protocol of a feasibility study
Source: BMJ Open. 2017 May 29;7(5):e014469. doi: 10.1136/bmjopen-2016-014469 (PMC5729976; doi:10.1136/bmjopen-2016-014469)
Supplement: Supplementary appendix 3 [file bmjopen-2016-014469supp003.pdf]

Appendix 3. Momentary and contextual questions

Momentary questions

1. I feel…
    a. Great
    b. Good
    c. Ok
    d. Not good
    e. Not good at all
2. How rested do I feel?
    a. Very rested
    b. Somewhat rested
    c. Neither rested nor tired
    d. Somewhat tired
    e. Very tired
3. How worried do I feel?
    a. Very worried
    b. Quite worried
    c. Somewhat worried
    d. A little worried
    e. Not at all worried
4. Am I enjoying myself?
    a. A lot
    b. Quite a lot
    c. Somewhat
    d. Not much
    e. Not at all
5. How energetic do I feel?
    a. Very energetic
    b. Fairly energetic
    c. Moderately energetic
    d. Not very energetic
    e. Not at all energetic

Contextual questions

1. Right now, I am…
    a. At home
    b. At work
    c. At the shops
    d. Outside
    e. At a Café or Restaurant
    f. In Transport
    g. At a Friend's or Family
    h. In a Clinic
    i. Other
2. Right now, I am…
    a. Relaxing

b. Working
c. Shopping
d. Looking After Others
e. Eating or Drinking
f. Travelling
g. Exercising
h. Waiting
i. None
