# Supplementary material for: A bespoke mobile application for the longitudinal assessment of depression and mood during pregnancy: protocol of a feasibility study
Source: BMJ Open. 2017 May 29;7(5):e014469. doi: 10.1136/bmjopen-2016-014469 (PMC5729976; doi:10.1136/bmjopen-2016-014469)
Supplement: Supplementary appendix 4 [file bmjopen-2016-014469supp004.pdf]

Appendix 4. Post-study acceptance survey

Please rate your agreement with the following statements with respect to this system:

1. This app is easy to use
    - Strongly agree – Strongly disagree (7-point Likert Scale)
2. I learned to use this app quickly
    - Strongly agree – Strongly disagree (7-point Likert Scale)
3. I would recommend this app to a friend
    - Strongly agree – Strongly disagree (7-point Likert Scale)
4. I would repeat the experience of using this app
    - Strongly agree – Strongly disagree (7-point Likert Scale)
5. I found using this app an engaging experience
    - Strongly agree – Strongly disagree (7-point Likert Scale)
6. I found the assessments useful
    - Strongly agree – Strongly disagree (7-point Likert Scale)
7. I was asked to provide reports:
    - Much too infrequently
    - Too infrequently
    - The right amount
    - Too frequently
    - Much too frequently
8. The experience of using this app met my needs:
    - Strongly agree – Strongly disagree (7-point Likert Scale)
9. What were your motivations in using this app?
10. What did you like the most about the experience of using this app?
11. What did you like least about the experience of using this app?
12. How would you improve the experience of using this app?
13. Do you have any other comments that you would like to make?

To be answered by those participants allocated to the retrospective plus momentary assessment strategy:

14. I found it useful to compare reports made right now with those made over the past 7 days:
    - Strongly agree – Strongly disagree (7-point Likert Scale)
