# Supplementary material for: A bespoke mobile application for the longitudinal assessment of depression and mood during pregnancy: protocol of a feasibility study
Source: BMJ Open. 2017 May 29;7(5):e014469. doi: 10.1136/bmjopen-2016-014469 (PMC5729976; doi:10.1136/bmjopen-2016-014469)
Supplement: Author's manuscript [file bmjopen-2016-014469.draft_revisions.pdf]

A bespoke mobile application for the longitudinal assessment of depression and mood during pregnancy: protocol of a feasibility study

Journal:	*BMJ Open*
Manuscript ID	bmjopen-2016-014469
Article Type:	Protocol
Date Submitted by the Author:	26-Sep-2016
Complete List of Authors:	Marcano Belisario, Jose Salvador; Imperial College London, Global eHealth Unit, Department of Primary Care and Public Health<br>Doherty, Kevin; University of Dublin Trinity College, School of Computer Science and Statistics<br>O'Donoghue, John; Imperial College London, Global eHealth Unit, Department of Primary Care and Public Health<br>Ramchandani, Paul; University of Oxford, Psychiatry<br>Majeed, Azeem; Imperial College, Primary Care<br>Doherty, Gavin; University of Dublin Trinity College, School of Computer Science and Statistics<br>Morrison, Cecily; Imperial College London, Global eHealth Unit, Department of Primary Care and Public Health<br>Car, Josip; Imperial College London, Global eHealth Unit, Department of Primary Care and Public Health
**Primary Subject Heading**:	Health informatics
Secondary Subject Heading:	Mental health, Obstetrics and gynaecology, Public health
Keywords:	Depression & mood disorders < PSYCHIATRY, Ecological momentary assessment, mHealth, Antenatal care, Mobile apps

SCHOLARONE™
Manuscripts

A bespoke mobile application for the longitudinal assessment of depression and mood during pregnancy: protocol of a feasibility study

Corresponding author

José S Marcano-Belisario

Room 304, Reynolds Building, St Dunstan's Road, London, W6 8RP, UK

jose.marcano-belisario10@imperial.ac.uk

Telephone: +44 (0)20 7594 0771

List of authors

José S Marcano-Belisario

Global eHealth Unit, Department of Primary Care and Public Health, School of Public Health, Imperial College London, London, UK

Kevin Doherty

School of Computer Science and Statistics, Trinity College Dublin, Dublin, Ireland

John O'Donoghue

Global eHealth Unit, Department of Primary Care and Public Health, School of Public Health, Imperial College London, London, UK

Paul Ramchandani

The Centre for Mental Health, Department of Medicine, Imperial College London, London, UK

Azeem Majeed

Department of Primary Care and Public Health, School of Public Health, Imperial College London, London, UK

Gavin Doherty

School of Computer Science and Statistics, Trinity College Dublin, Dublin, Ireland

Cecily Morrison

Global eHealth Unit, Department of Primary Care and Public Health, School of Public Health, Imperial College London, London, UK

Josip Car

Global eHealth Unit, Department of Primary Care and Public Health, School of Public Health, Imperial College London, London, UK

For peer review only

Keywords

Perinatal depression

Mood

Ecological momentary assessment

Edinburgh Postnatal Depression Scale

Mobile phones

mHealth

Word count

3820 words

For peer review only

ABSTRACT

Introduction

Depression is one of the most common mental health disorders during pregnancy, with important consequences for mothers and their children. Despite this, it goes undiagnosed and untreated in many women attending antenatal care. Smartphones could help support the prompt identification of antenatal depression. In addition, these devices enable the implementation of ecological momentary assessment techniques, which could provide a more accurate picture of how mood and depression are experienced during pregnancy. With this study, we will assess the feasibility of using a bespoke mobile application running on participants' own handsets for the longitudinal (6 months) screening and monitoring of antenatal mood and depression.

Methods and analysis

We will use a randomised controlled study design to compare two types of assessment strategies: retrospective + momentary (consisting of the Edinburgh Postnatal Depression Scale plus 5 momentary and 2 contextual questions), and retrospective (consisting of the Edinburgh Postnatal Depression Scale only). We will assess the impact that these strategies have on participant adherence to a pre-specified sampling protocol, drop-out rates and timeliness of data completion. We will also evaluate differences in acceptance of the technology, as well as the potential effect that momentary assessments could have on retrospective data. We will attempt to identify any patterns in app usage through the analysis of log data.

Ethics and dissemination

This study has been approved by the South East Coast – Surrey Research Ethics Committee. Our findings will be disseminated through academic peer-reviewed publications, conferences and discussion with peers.

Registration details

This study has been registered in ClinicalTrials.gov under the identifier NCT02516982.

For peer review only

STRENGTHS OF THIS STUDY

- This study explores the role of mobile technology as a medium to (i) address some of the barriers preventing depression screening in antenatal settings, and to (ii) evaluate how mood and depression are experienced throughout pregnancy (using momentary, experiential and ecologically valid data)
- This study will assess two critical success factors for the successful deployment of mobile technology in antenatal settings, namely, participant engagement with, and acceptance of, the technology and adherence to a proposed sampling protocol
- This study will provide baseline information regarding the appropriateness of a sampling protocol (in terms of its duration, intensity and frequency) for the monitoring of mood and depression during the antenatal period, through a combination of retrospective and momentary assessments
- The results from this study could inform similar strategies for the assessment of other common mental health problems in pregnancy and their risk factors, as well as for the collection of routine antenatal care data
- The technology used in this study has been specifically designed and developed to fit within the clinical context and the local pathways in which it will be deployed

LIMITATIONS OF THIS STUDY

- This study focuses on antenatal depression alone. However, anxiety and post-traumatic stress disorder are also common mental health disorders that occur during pregnancy
- The app developed for this study focuses on the assessment of mood and symptoms of clinical depression, and will be deployed alongside existing patient-midwife relationships. However, one of the key elements in mental health research is being able to gather information about potential triggers or risk factors (e.g., domestic violence)
- This study relies solely on self-report measures. Research is being conducted to explore how data that are routinely collected by smartphones and app usage could be used to unobtrusively identify low mood and depression in the general population. These types of analysis are not without challenges; however, they could circumvent some of the disadvantages of self-report measures

For peer review only

INTRODUCTION

Antenatal depression is one of the most common, treatable mental health disorders in pregnancy.[1-4] Point prevalence estimates vary between 7% and 12% (depending on the trimester), and period prevalence estimates suggest that as many as 12.7% of pregnant women could experience an episode of major depression.[3,5] Moreover, antenatal depression is associated with long-term adverse health outcomes in both mothers and their offspring. Pregnant women suffering from depression are more likely to engage in unhealthy practices (including poor diet, substance abuse, and failure to enroll in prenatal care), and are at increased risk of self-harm (or suicide) and postpartum depression.[1,6] Antenatal depression can also affect foetal development, and has been identified as an independent risk factor for a child's behavioural, cognitive and emotional development (including through adolescence).[1,7-9]

Despite the availability of effective therapeutic options, antenatal depression goes undiagnosed and untreated in many cases, with up to three-quarters of pregnant women meeting diagnostic criteria for depression (and anxiety) not identified, and only 1 in 10 of those who require further treatment are able to access it.[10] Some of the barriers to the prompt diagnosis of this disorder include difficulties in differentiating depressive symptoms from the expected mood and somatic changes of pregnancy; stigma; lack of reassurance that mental health care is a normal part of antenatal care; characteristics of healthcare providers; configuration of health services; insufficient consultation time; and the cost-effectiveness of screening practices.[10-12]

As with other mental health conditions,[13-16] smartphones could help address some of these barriers and facilitate the screening and monitoring of depression throughout the antenatal period. The computational capabilities of these devices allow them to implement validated screening scales (usually retrospective self-reports) at any frequency and for any duration. Smartphones are also able to support the implementation of techniques for the collection of momentary, experiential and ecologically valid data. Being collected in real time, momentary data are less susceptible to many of the biases common to retrospective scales (e.g., recall bias), and are more sensitive to fluctuations over time.[17-19] The combination of these two types of data could provide a more accurate picture of antenatal depression. The networking capabilities and wide availability (approximately 71% of UK

For peer review only

adults)[20] of smartphones, and the software development and distribution framework of mobile applications (apps), could help practitioners to circumvent some of the practical challenges associated with screening and clinical monitoring, and reduce the costs associated with data handling and management.

Altogether, these characteristics could contribute to making the screening and monitoring of antenatal depression more cost effective: an initial resource-intensive app development phase would be followed by a relatively low-cost, large scale distribution of the app onto patients own smartphones. Thereafter, regular depression assessments could take place remotely, at anytime and anywhere (with the provision of treatment being dependent upon the configuration of local healthcare services). Nonetheless, the feasibility of using smartphones for this purpose needs to be explored.

In this area of research, a key factor is patients' willingness to run screening or clinical monitoring apps on their personal handsets.[16] This could influence patient compliance with clinician-led data gathering (sampling) protocols, and thus affect data completeness. The latter refers to the minimum amount of information required by clinicians to inform their decisions, and is an important data quality dimension in healthcare.[21] Data completeness is also susceptible to the burden that the intensity of sampling protocols (both in terms of frequency and duration) can place on patients. Moreover, the impact that adding momentary assessments can have on retrospective data also needs to be explored, as this could lead to more efficient diagnostic and therapeutic decisions.

This study is part of a project aimed at understanding the role of mobile technology for the screening and assessment of antenatal depression and psychological wellbeing. A previous feasibility study assessed the feasibility of using iPads in the waiting area of antenatal clinics for implementing *The National Institute for Health and Care Excellence* (NICE) recommendations for the recognition of depression.[22] The present study will explore the feasibility of using a bespoke app to support the longitudinal and remote assessment of mood and depression throughout the antenatal period. In order to evaluate issues of patient acceptance and data quality, we will compare two 6-month sampling protocols requiring either (i) monthly retrospective *and* momentary assessments or (ii) monthly retrospective assessments.

For peer review only

METHODS AND ANALYSIS

study design

We will assess the feasibility of using a bespoke mobile application, called BrightSelf, running on participants' own smartphones to assess and monitor depression and mood during the antenatal period through a combination of retrospective assessments and ecological momentary assessments (EMA).

We will use a parallel, randomised controlled study design to assign our participants to one of two types of assessment strategies:

1. Retrospective plus momentary assessment: requiring the completion of (i) the Edinburgh Postnatal Depression Scale (EPDS), (ii) 5 momentary questions (assessing a participant's mood, sleep, worry, enjoyment and energy levels), and (iii) 2 contextual questions at irregular intervals once a month for 6 months; or
2. Retrospective assessment: requiring the completion of the EPDS at irregular intervals once a month for 6 months.

sample selection and recruitment

We will select our sample of participants from pregnant women in their first trimester who are attending antenatal clinics in general practices, community services, and secondary care NHS centres in England.

On the day of their antenatal appointment, each potential participant will be approached by a clinical studies officer (CSO) or a research midwife with appropriate good clinical practice (GCP) training, and will be provided with a participant information sheet. Potential participants expressing their interest in taking part in this study, will be assessed against our inclusion and exclusion criteria (Table 1).

Table 1. Participant inclusion and exclusion criteria

INCLUSION CRITERIA	EXCLUSION CRITERIA
Women who are 18 years old or older	Current diagnosis of depression or other mood disorder made by a health professional
In the first trimester of pregnancy at the time of consent	Currently receiving treatment for depression or other mood disorder

For peer review only

Any parity	Recent personal history of depression or other mood disorder in the past 12 months
Attending antenatal clinics in participating NHS sites	Not comfortable reading and writing in English
Own smartphone (either an iPhone or any type of Android handset)	Not owning a smartphone, or owning an incompatible handset [i.e., Windows Phone, Blackberry, or Linux]

Potential participants meeting our inclusion criteria will have all the study details explained to them, and will be given the opportunity to ask as many questions about the study as they need. Potential participants will have at least 24 hours to decide on participation; refusal to take part in this study will not have an impact on their legal rights, medical care or their relationship with care providers.

We will obtain written, informed consent from those potential participants who, after receiving all the relevant study information and having all their questions answered to their satisfaction, still wish to take part in this study.

After obtaining consent, participants will be asked to complete a baseline assessment consisting of a (i) socio-demographic survey, (ii) the Whooley questions, and (iii) the EPDS (Appendix 1). Subsequently, the CSO or research midwife will guide participants through the process of downloading (from either the Apple AppStore or Google Play Store) and installing BrightSelf onto their own handsets. To this end, they will be able to use a recruiter booklet provided by the central research team (Appendix 2). In order to activate the app, participants will need to enter a 9-digit activation code, which will also be provided by the central research team.

interventions to be measured

Surveys

Non-validated, socio-demographic survey

We will administer an 11-question survey to collect information about participants' age group, ethnic background, marital status, employment status, level of education, smartphone and tablet computer ownership, obstetric history, and previous personal history of depression.

For peer review only

Whooley questions

The Whooley questions were developed as a case-finding instrument for depression in primary care.[23] This 2-question instrument assesses depressed mood and anhedonia that have been present during the past month. Respondents are required to answer *Yes* or *No* to each question.

Edinburgh Postnatal Depression Scale

The EPDS is a 10-item self-administered survey that was developed to screen for perinatal depression in the community.[24] This instrument assesses feelings of guilt, sleep disturbance, reduced energy levels, anhedonia and suicidal ideation that have been present during the past 7 days. Each question is scored on a 4-point scale ranging from 0 to 3 points.

An overall score generated from the sum of these responses. Overall scores between 10 and 12 points suggest increased risk for depression; scores of 13 points or more suggest that the diagnostic criteria for major depression disorder have probably been met.[25] In addition, special attention should be paid to item 10, as it deals with suicidal thoughts. Based on these scores, a clinician would be prompted to refer a woman to a mental health professional.

The EPDS is a valid and reliable tool for identifying women who are at risk of depression, both during pregnancy and postpartum. This instrument is also sensitive to changes in the severity of depression over time.[24] The EPDS can be reproduced without further permission provided that the original source of the scale is cited in each reproduced copy.

Non-validated, momentary mood questions

We will administer 5 momentary questions to assess participants' mood, sleep, worry, enjoyment and energy (Appendix 3). These questions are based on the work of a research fellow at the Collaboration for Leadership and Applied Health Research and Care (CLAHRC) for the East of England.[26] Each question will be mapped onto 5-point pictorial scales, ranging from 1 (low) to 5 points (high). For the purpose of this feasibility study, we will not perform any overall score calculation or attempt any validation of these questions.

Non-validated, contextual questions

For peer review only

Two contextual questions will complement the momentary mood questions (Appendix 2). They will assess (i) participants' location and (ii) the activity in which they were engaged at the time they were required to complete the 5 momentary mood questions.

Non-validated, post-study acceptance survey

We will administer 13 questions (to participants completing retrospective assessments) or 14 questions (to participants completing retrospective plus momentary assessments) at the end of the 6-month participation period (Appendix 4). The purpose of these questions is to assess the acceptability to participants of BrightSelf in the context of their antenatal care, and to gather information about their experience of using it.

Mobile Application

A bespoke mobile application called BrightSelf will be used in this study. This app is compatible with the iPhone Operating System (iOS) developed by Apple Inc and with the Android Operating System developed by Google. A description of the design and development of BrightSelf, and a detailed account of its functionality is the purpose of another publication.

Here we present the two features that are most relevant to this feasibility study.

Check Back

This feature will enable the administration of the EPDS. It consists of two introductory screens informing participants of the retrospective nature of this scale, and its intended applications. These screens will be followed by the 10 EPDS questions, presented one question per screen, using radio buttons to capture participants' responses (Figure 1).

Check In

This feature will enable the administration of the 5 momentary questions and the 2 contextual questions. The first 5 questions will be presented using a 5-point pictorial scale with temporary supporting text (see Figure 2 for an example). The last 2 questions will use radio buttons to capture participants' responses.

Retrospective versus momentary assessments

Participants will be asked to complete monthly assessments at irregular intervals for 6 months. Whenever an assessment is due (as per the sampling protocol), a notification will

For peer review only

be displayed on a participant's handset. There will be two types of notifications: one for the *Check Back* feature and one for the *Check In* feature of BrightSelf. The visual appearance of the notifications will depend on the operating system and on the model of the participant's handset. The text of the notifications however, will remain constant across participants for each type of notification.

Participants will be able to respond (and thus complete the corresponding assessments) or to dismiss the notifications. In addition, participants will be able to leave notifications unanswered. If participants do not complete an assessment in response to a notification, they will not receive reminders or follow-up notifications. In these instances, the non-response will be recorded in our database and will be coded as such for the purposes of our data analysis.

Outside assessment periods, participants will be able to use BrightSelf in any way they find convenient and there will be no restrictions on the number of assessments they can complete on a single day. However, we will be able to distinguish between assessments completed in response to a notification and those completed spontaneously. In either situation, the research team will be alerted if any of a series of pre-specified conditions are met; alerts will be colour-coded according to their severity (Table 2). The research team will then notify the corresponding clinical teams.

Table 2. Criteria for an alert to be sent to the research team

TYPE OF ALERT	CRITERIA
Yellow [Mild]	Completing more than one EPDS assessment on the same day, with an overall EPDS score of 9 points or less and a score of 0 on question 10 of the EPDS
Orange [Moderate]	Overall EPDS score between 10 and 12 points, with a score of 0 on question 10 of the EPDS
Red [Severe]	Overall EPDS score of 13 points or more<br><br>1 or more points on question 10 of the

For peer review only

	EPDS, regardless of the overall EPDS score

At baseline, participants will be randomly allocated to one of two types of assessment strategies:

Retrospective plus Momentary Assessments

In this experimental manipulation, participants will be asked to complete a combination of retrospective assessments and momentary assessments. A retrospective assessment will be defined as a single administration of the EPDS. A momentary assessment will be defined as the 5 momentary questions plus the 2 contextual questions.

Participants will be required to complete assessments for a total of 6 months, and each assessment period will consist of 6 consecutive days. The 6-month participation period will be calculated from the day on which the app is activated (i.e., when participants enter their activation code and activate their account), and it will be divided into six intervals. All participants will receive the first notification 2 days after activating BrightSelf (i.e., first assessment), while the use of the app is fresh within their minds and to give an idea of what to expect during the study. Subsequent assessment periods will be a random selection of 6 consecutive days within the 21 to 35 days following the end of the previous assessment period (in order to avoid 12 days of continuous assessments).

The assessment period will be structured as follows:

- Day 1: one retrospective assessment at any random time between 17:00 and 21:00;
- Day 2 to 5: 3 momentary assessments per day, displayed at random times within each of the following intervals: 09:00 – 12:00; 13:00 – 16:00; and 17:00 – 20:00; and
- Day 6: one retrospective assessment at any random time between 17:00 and 21:00.

At the end of the 6-month period, participants will be sent a link via short messaging service (SMS) or by email to complete the non-validated, post-study acceptance survey.

Retrospective Assessments

In this experimental manipulation, participants will be asked to complete retrospective assessments only. A retrospective assessment will be defined as a single administration of the EPDS.

For peer review only

Participants will be required to complete assessments for a total of 6 months, and each assessment period will consist of one day. The 6-month participation period will be calculated from the day on which the app is activated (i.e., when participants enter their activation code and activate their account), and it will be divided into six intervals. Again, all participants will receive the first notification 2 days after activating BrightSelf (i.e., first assessment). Subsequent assessments will take place on a random day within the 21 to 35 days following the end of the previous assessment period (in order to avoid two consecutive EPDS assessments).

On each assessment day, participants will receive a notification to complete the EPDS at a random time between 17:00 and 21:00.

At the end of the 6-month period, participants will be sent a link via SMS or by email to complete the non-validated, post-study acceptance survey.

See Figure 3 for an illustration of both types of assessment.

randomisation

We will use block randomisation procedures (with blocks of 4) to allocate participants to one of the two experimental arms. Random numbers will be generated using Stata 14.[27] Each consecutive number will be embedded within the 9-digit activation codes that will be distributed to each participating NHS sites. The full activation codes will be generated in the same order in which the random numbers were generated. Each NHS site will receive a list of activation codes on a first-come-first-served basis. Members of the local research teams will not be informed of the random sequence generation and will not be able to identify which number determines participant allocation. They will be required to use activation codes sequentially, as participants are recruited. Activation codes referring to the *Retrospective plus momentary assessment* condition will activate a version of BrightSelf in which both the *Check Back* and the *Check In* features are active. Activation codes referring to the *Retrospective assessment* condition will activate a version of BrightSelf in which only the *Check Back* feature is active.

outcomes

Adherence to sampling protocols

For peer review only

We will calculate the number of participants who complete 100% of the expected assessments (as per the study protocol) as a proportion of the total number of participants who were randomised into the study. We will sub-divide this outcome by using the total number of participants who complete the 6-month participation period as the denominator.

Drop-out rates

We will calculate the number of participants who complete the 6-month participation period as a proportion of the total number of participants who were randomised into the study.

Usage patterns

We will assess participants' usage pattern of BrightSelf by analysing log data (additional variables capturing app usage). These will include the number of additional voluntary self-reports, time spent using the app, number of interactions with other sections of the app, time taken to complete the self-assessments, and other ancillary usage data.

Acceptance

We will calculate participants' ratings and responses to the post-study acceptance survey.

Timeliness of data completion

We will calculate the number of assessments that were completed in response to a notification, as a proportion of the total number of expected assessments. We will sub-divide this outcome by using the total number of completed assessments as the denominator. We will consider that an assessment has been completed in response to a notification if it takes place within the interval corresponding to that notification (i.e., before the next notification is delivered). This broad interval may raise concerns regarding the ecological validity of the report, which we will explore in our statistical analysis.

sample size calculations

We have chosen to relate the proposed sample size to the 95% confidence interval for the adherence rate, as recommended by the Research Design Service in London. Therefore, we would need 96 participants in each arm with a 95% confidence level and a confidence interval of 10. This translates into a total sample of 192 (i.e., 96 participants in each experimental group). For this reason, we will aim to recruit at least 200 participants, to

For peer review only

account for drop-outs.

data analysis plan

Descriptive statistics

We will report the number of potential participants who were eligible and refused to take part in the study. Where possible, we will report the reasons for refusing to participate.

For each experimental group, we will report the following information:

- Demographic characteristics (as captured by the non-validated, socio-demographic survey);
- Proportion of participants answering Yes to any of the Whooley questions during the baseline assessment;
- Proportion of participants scoring at each interval of the EPDS: between 0 and 9 points; between 10 and 12 points; and 13 points or above during the baseline assessment; and
- Proportion of participants scoring 1 or more points on question 10 of the EPDS during the baseline assessment

Inferential statistics

We will compare the *Retrospective and Momentary assessment* and the *Retrospective assessment* experimental groups for differences in adherence rates, drop-out rates and timeliness of data completion. For this we will use a t-test or the non-parametric equivalent.

We will compare acceptance between the two experimental manipulations by assessing differences in participants' rankings to those questions on a 7-point Likert scale of the post-study acceptance survey. We will also conduct a thematic analysis of the open-ended questions of this survey.

In relation to the momentary assessments, we will examine the distribution of delays between the time of notification and time of report in order to assess the effect of this delay on the ecological validity of reports.

We will analyse usage patterns through regression modelling of log data. We will analyse participants momentary and retrospective assessments through time-based analyses or multi-level modelling.[28,29] We will attempt to compare if the momentary assessments

For peer review only

had any effect on the retrospective assessments by comparing the EPDS responses given on Day 1 to those given on Day 6.

timeline

We expect participant recruitment to start in October 2016, and end in December 2016. We expect the last follow up to take place in June 2017.

For peer review only

CONCLUSION

This study addresses an important area of unmet clinical need, with direct and indirect consequences for mothers, children, their families, health systems and society. This study will contribute to the growing body of evidence concerning the role of mobile technologies for the support of mental health. Similarly, it will generate baseline information concerning the acceptability of an EMA sampling protocol (in term of its duration, frequency and intensity) to women attending antenatal care. This study will also evaluate participant engagement with the technology and their adherence to a pre-specified sampling protocol; both of which influence the completeness of the data needed by clinicians to inform their decisions. In addition, this study will identify some of the implementation issues that might arise when we attempt to deploy mobile technologies in clinical settings, which could affect their successful adoption.

In this work, we have maintained a focus on antenatal depression. Mental health during pregnancy however, is more complex. Disorders such as anxiety and post-traumatic stress disorder are also amongst the most common disorders during this period. Moreover, pregnant women who are exposed to certain risk factors or triggers (e.g., domestic violence, social isolation, minority groups, and low-income) are at increased risk of suffering from any of these mental health problems. Although we have not been able to focus on all these issues, we believe that the findings from this feasibility study will produce important lessons that could enable us to design and develop similar technologies and appropriate strategies. We aim to recruit participants from diverse backgrounds, and across multiple settings and geographical areas within England, and to focus on how such technologies can be embedded within existing antenatal care pathways, in order to support the existing patient-midwife relationship.

The findings from this and from a previous feasibility study[19] will inform a larger trial evaluating the integration of mobile technologies into routine antenatal pathways, and their potential impact on clinical outcomes.

For peer review only

ETHICS AND DISSEMINATION

This study has been reviewed and approved by the National Research Ethics Service (NRES) Committee South East Coast – Surrey on 15 April 2016 as a notice of substantial amendment to the original submission (09 July 2015) under the Research Ethics Committee (REC) reference 15/LO/0977.

This study is being sponsored by Imperial College London under the reference number 15IC2687 and has been included in the UK Clinical Research Network (CRN) Study Portfolio under the Central Portfolio Management System (CPMS) number 19280.

Lastly, this study protocol has been registered in ClinicalTrials.gov under the identifier NCT02516982 (as required by the REC).

The findings of this study will be disseminated through academic peer-reviewed publications, poster presentations and abstracts at academic and professional conferences, discussion with peers, and social media. The findings of this study will also inform the PhD theses of Jose Marcano Belisario and Kevin Doherty.

For peer review only

REFERENCES

1 Stewart DE. Depression during pregnancy. N Engl J Med 2011;365:1605-11.

2 Mental Health Foundation. Fundamental Facts About Mental Health 2015. London: Mental Health Foundation 2015.

3 Gavin NI, Gaynes BN, Lohr KN, et al. Perinatal depression: a systematic review of prevalence and incidence. Obstet Gynecol 2005;106(5 Pt 1):1071-83 doi:10.1097/01.AOG.0000183597.31630.db.

4 Lee AM, Lam SK, Sze Mum Lau SM, et al. Prevalence, course, and risk factors for antenatal anxiety and depression. Obstet Gynecol 2007;110(5):1102-12 doi:10.1097/01.AOG.0000287065.59491.70

5 Bennett HA, Einarson A, Taddio A, et al. Prevalence of depression during pregnancy: systematic review. Obstet Gynecol 2004;103(4):698-709 doi:10.1097/01.AOG.0000116689.75396.5f.

6 Heron J, O'Connor TG, Evans J, et al. The course of anxiety and depression through pregnancy and the postpartum in a community sample. J Affect Disord 2004;80(1):65-73 doi:10.1016/j.jad.2003.08.004

7 Kinsella MT, Monk C. Impact of Maternal Stress, Depression & Anxiety on Fetal Neurobehavioral Development. Clin Obstet Gynecol 2009;52(3):425-440 doi:10.1097/GRF.0b013e3181b52df1.

8 Pearson RM, Evans J, Kounali D, et al. Maternal Depression During Pregnancy and the Postnatal Period: Risks and Possible Mechanisms for Offspring Depression at Age 18 Years. JAMA Psychiatry 2013;70(12):1312-9 doi:10.1001/jamapsychiatry.2013.2163.

9 Braithwaite EC, Murphy SE, Ramchandani PG. Effects of prenatal depressive symptoms on maternal and infant cortisol reactivity. Arch Womens Ment Health 2016;19:581-590 doi:10.1007/s00737-016-0611-y

10 Kingston D, Austin MP, Heaman M, et al. Barriers and facilitators of mental health screening in pregnancy. J Affect Disord 2015;186:350-7 doi:10.1016/j.jad.2015.06.029 [published Online First: 29 July 2015].

For peer review only

11 Goodman JH. Women's attitudes, preferences, and perceived barriers to treatment for perinatal depression. Birth 2009;36(1):60-9 doi:10.1111/j.1523-536X.2008.00296.x.

12 Paulden M, Palmer S, Hewitt C, et al. Screening for postnatal depression in primary care: cost effectiveness analysis. *BMJ* 2009;339:b5203 doi:http://dx.doi.org/10.1136/bmj.b5203 [published Online First: 23 December 2009].

13 BinDhim NF, Alanazi EM, Aljadhey H, et al. Does a Mobile Phone Depression-Screening App Motivate Mobile Phone Users With High Depressive Symptoms to Seek a Health Care Professional's Help? J Med Internet Res 2016;18(6):e156 doi:10.2196/jmir.5726.

14 BinDhim NF, Shaman AM, Trevena L, et al. Depression screening via a smartphone app: cross-country user characteristics and feasibility. J Am Med Inform Assoc 2015;22(1):29-34 doi:10.1136/amiajnl-2014-002840 [published Online First: 17 October 2014]

15 Woo YS, Bahk WM, Hong J, et al. Use of a smartphone application to screen for bipolar sprectrum disorder in a community sample. Health Informatics J 2016;22(3):779-88 doi:10.1177/1460458215589601 [published Online First: 17 July 2015].

16 Torous J, Chan SR, Yee-Marie Tan S, et al. Patient Smartphone Ownership and Interest in Mobile Apps to Monitor Symptoms of Mental Health Conditions: A Survey in Four Geographically Distinct Psychiatric Clinics. JMIR Mental Health 2014;1(1):e5 doi:10.2196/mental.4004.

17 Moskowitz DS, Young SN. Ecological momentary assessment: what it is and why it is a method of the future in clinical psychopharmacology. J Psychiatry Neurosci 2006;31(1):13-

18 Shiffman S, Stone AA, Hufford MR. Ecological momentary assessment. Annu Rev Clin Psychol 2008;4:1-32.

19 Asselbergs J, Ruwaard J, Ejdys M et al. Mobile Phone-Based Unobtrusive Ecological Momentary Assessment of Day-to-Day Mood: An Explorative Study. J Med Internet Res 2016;18(3):e72 doi:10.2196/jmir.5505.

20 Ofcom. Facts & Figures. http://media.ofcom.org.uk/facts/ [date accessed: 30 August 2016].

21 Marcano-Belisario JS, Jamsek J, Huckvale K, et al. Comparison of self-administered survey questionnaire responses collected using mobile apps versus other methods. Cochrane

For peer review only

Database Syst Rev 2015;27(7):MR000042 doi:10.1002/14651858.MR000042.pub2 [published Online First: 27 July 2015].

22 Marcano-Belisario JS, Gupta AK, O'Donoghue J, et al. Tablet computers for implementing NICE antenatal mental health guidelines: protocol of a feasibility study. BMJ Open 2016;6:e009930 doi:10.1136/bmjopen-2015-009930 [published Online First: 22 January 2016].

23 Whooley MA, Avins AL, Miranda J, et al. Case-finding instruments for depression – Two questions are as good as many. J Gen Intern Med 1997;12(7):439-45.

24 Cox JL, Holden JM, Sagovsky R. Detection of Postnatal Depression - Development of the 10-Item Edinburgh Postnatal Depression Scale. Br J Psychiatry 1987;150:782-86.

25 Allbaugh LJ, Marcus SM, Ford EC, et al. Development of a screening and recruitment registry to facilitate perinatal depression research in obstetrics settings in the USA. Int J Gynaecol Obstet 2015;128(3):260-63 doi:10.1016/j.ijgo.2014.09.015 [published First Online: 11 November 2014].

26 Blake F. Mumshine – The App: The design of a micro on-line intervention for postnatal women to improve mood and well-being using an app for the smart phone. Presented at: CLAHRC EAST OF ENGLAND FELLOWS SHOWCASE EVENT. 4 December 2014.

27 Stata. Stata Statistical Software [Program]. Version 14, 2015.

28 Schwartz JE, Stone AA. Strategies for analyzing ecological momentary assessment data. Health Psychol 1998;17(1):6-16.

29 Shiffman S. Conceptualizing Analyses of Ecological Momentary Assessment Data. Nicotine Tob Res 2014;16(Suppl 2):S76-S87 doi:10.1093/ntr/ntt195 [published First Online: 9 December 2013].

For peer review only

AUTHORS' CONTRIBUTIONS

José S Marcano-Belisario (JMB) developed the study protocol in collaboration with Kevin Doherty (KD). JMB was in charge of obtaining ethics and governance approvals, and has been approaching potential participant recruitment centres. KD was in charge of software development. JMB drafted this manuscript. Cecily Morrison (CM), Gavin Doherty (GD), Paul Ramchandani (PR), John O'Donoghue (JOD), Azeem Majeed (AM), and Josip Car (JC) guided the development of the study protocol. CM, JOD and JC have supervised JMB's work. GD has supervised KD's work. All the authors reviewed and approved this manuscript.

ACKNOWLEDGEMENTS

We would like to thank Prof Susan Ayers and Alexandra Thornton at City University London, Joanna Girling and Marie O'Connell at West Middlesex Hospital, Tara Pauley and Charlotte Clayton at Hinchingbrooke Hospital, Antoinette McNulty and Gemma Loebenberg at the Northwest London CRN, and Dr Fiona Blake for their feedback on early versions of BrightSelf and the study protocol, and for their help with our patient and public involvement initiatives. Thank you to Marguerite Barry at Trinity College Dublin for running some of the patient and public involvement sessions with us.

FUNDING STATEMENT

This work is being supported by a National Institute for Health Research (NIHR) Imperial Biomedical Research Centre (BRC) award through the Population Health Theme, and by the ADAPT Centre through Trinity College Dublin.

COMPETING INTERESTS STATEMENTS

The authors declare that they do not have any competing interests.

For peer review only

LIST OF FIGURES

Figure 1. BrightSelf – Check Back (screenshot)

Figure 2. BrightSelf – Check In (screenshot)

Figure 3. Sampling protocols for the retrospective plus momentary assessments and for the retrospective assessments

For peer review only

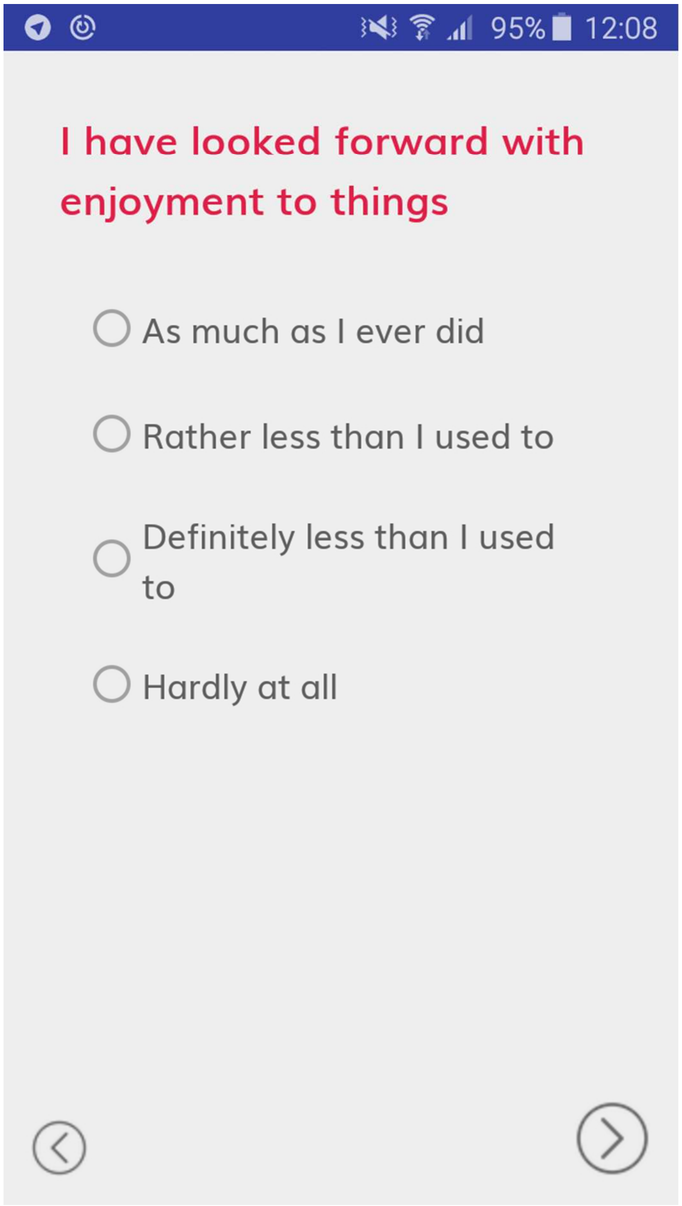

Figure 1. BrightSelf – Check Back (screenshot)

254x451mm (72 x 72 DPI)

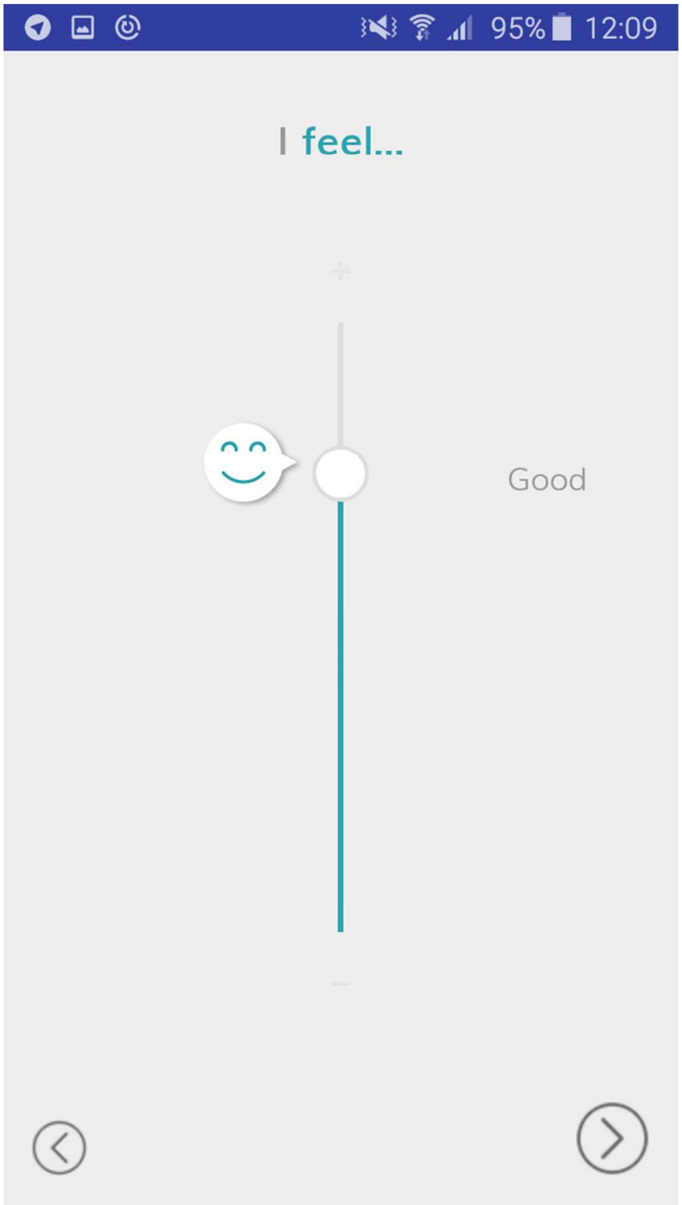

Figure 2. BrightSelf – Check In (screenshot)

254x451mm (72 x 72 DPI)

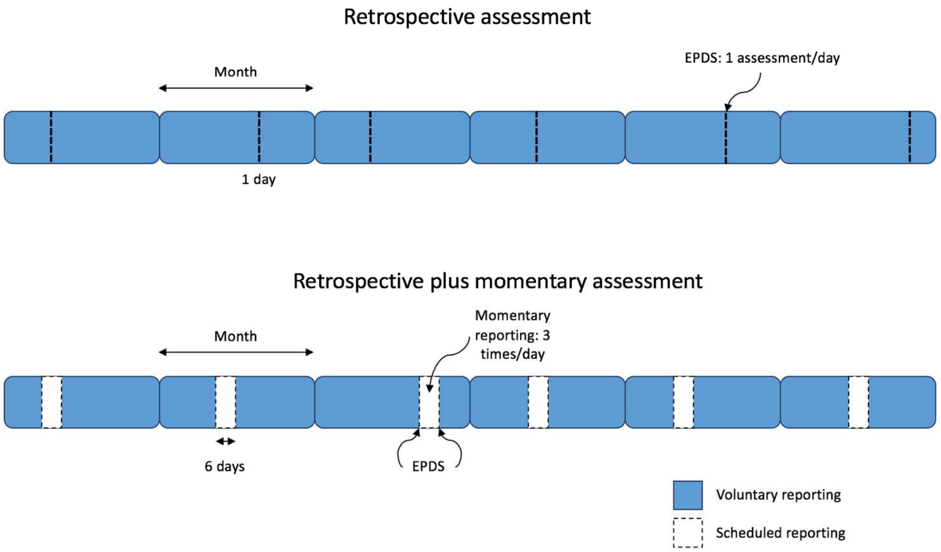

Figure 3. Sampling protocols for the retrospective plus momentary assessments and for the retrospective assessments

Appendix 1. Baseline assessments

Socio-demographic survey

- What is your age group?
  - 18 to 22 years
  - 23 to 27 years
  - 28 to 32 years
  - 33 to 37 years
  - 38 years or older
- How would you describe your race or ethnicity?
  - White
    - British
    - Irish
    - Other
  - Mixed
    - White and Black Caribbean
    - White and Black African
    - White and Asian
    - Other
  - Asian or Asian British
    - Indian
    - Pakistani
    - Bangladeshi
    - Other
  - Black or Black British
    - Caribbean
    - African
    - Other
  - Chinese
  - Other
  - Prefer not to say
- What is your relationship status?
  - Single
  - Married/In a civil partnership
  - Living with partner
  - Divorced/Civil partnership that has been dissolved
  - Widowed
  - Separated
  - Prefer not to say
- What is your employment status?
  - Employed, full-time
  - Employed, part-time
  - Self-employed
  - Not employed, looking for work
  - Not employed, not looking for work
  - Disability/Not able to work

For peer review only

- What is your highest level of education?
  - Postgraduate – PhD/Doctorate
  - Postgraduate – Masters degree
  - University or college degree
  - University or college qualification below degree level
  - A Levels or equivalent
  - GCSE or equivalent
  - Other
  - None of these
- Do you own a smartphone (for example, an iPhone or Samsung phone)?
  - Yes
  - No
- Do you own a tablet computer (for example, an iPad)?
  - Yes
  - No
- How many times have you been pregnant (please count your current pregnancy)?
  - 1
  - 2
  - 3
  - 4 or more
- How many children have you given birth to?
  - 0
  - 1
  - 2
  - 3 or more
- When is your baby due? (please indicate month and year)
- Have you ever been diagnosed with depression?
  - Yes
  - No

Whooley questions

- Over the past month, have you been bothered by feeling down, depressed or hopeless?
  - Yes
  - No
- Over the past month, have you been bothered by having little interest or pleasure in doing things?
  - Yes
  - No

Edinburgh Postnatal Depression Scale

As you are pregnant or have recently had a baby, we would like to know how you are feeling. Please check the answer that comes closest to how you have felt IN THE PAST 7 DAYS, not just how you feel today.

In the past 7 days:

For peer review only

1. I have been able to laugh and see the funny side of things
    a. As much as I always could
    b. Not quite so much now
    c. Definitely not so much now
    d. Not at all
2. I have looked forward with enjoyment to things
    a. As much as I ever did
    b. Rather less than I used to
    c. Definitely less than I used to
    d. Hardly at all
3. I have blamed myself unnecessarily when things went wrong
    a. Yes, most of the time
    b. Yes, some of the time
    c. Not very often
    d. No, never
4. I have been anxious or worried for no good reason
    a. No, not at all
    b. Hardly ever
    c. Yes, sometimes
    d. Yes, very often
5. I have felt scared or panicky for no very good reason
    a. Yes, quite a lot
    b. Yes, sometimes
    c. No, not much
    d. No, not at all
6. Things have been getting on top of me
    a. Yes, most of the time I haven't been able to cope at all
    b. Yes, sometimes I haven't been coping as well as usual
    c. No, most of the time I have coped quite well
    d. No, I have been coping as well as ever
7. I have been so unhappy that I have had difficulty sleeping
    a. Yes, most of the time
    b. Yes, sometimes
    c. Not very often
    d. No, not at all
8. I have felt sad or miserable
    a. Yes, most of the time
    b. Yes, quite often
    c. Not very often
    d. No, not at all
9. I have been so unhappy that I have been crying
    a. Yes, most of the time
    b. Yes, quite often
    c. Only occasionally
    d. No, never
10. The thought of harming myself has occurred to me
    a. Yes, quite often
    b. Sometimes
    c. Hardly ever
    d. Never

For peer review only

For peer review only

BRIGHTSELF

Welcome Guide (Arm 2)

Version1.0 - 6 Jul 2016

Welcome

Hello, and thank you for collaborating with this study to test the feasibility of BrightSelf, an application for the assessment of psychological wellbeing during pregnancy.

This application and study are the result of a collaboration between Imperial College London and Trinity College Dublin.

Here you will find a quick explanation of BrightSelf, the value it provides to participants, and guidelines for recruitment.

The following section answers any questions the user may have and might be of use to you during recruitment.

For peer review only

Questions Answered

What is BrightSelf?

BrightSelf is a new mobile app designed to help women develop a picture of their psychological wellbeing during pregnancy.

Researchers at Imperial College London and Trinity College Dublin are currently assessing the engagement of mothers-to-be with BrightSelf. In future, we hope to assess the value of the app to improve the wellbeing of mothers-to-be and communication between women and their healthcare providers.

By **checking in** you can capture your mood, sleep, energy and more in the moment. By **checking back**, BrightSelf allows you to build a picture of your wellbeing over the past week. BrightSelf also provides the ability to interact with your data, information on wellbeing and research, sources of professional support, and an ideas machine, your personal source for a pick-me-up!

For peer review only - http://bmjopen.bmj.com/site/about/guidelines.xhtml

What is expected of users?

BrightSelf is here for you to use in any way you find useful. You can make self reports according to a schedule you determine for yourself or simply as often as you feel useful. We have tried to design BrightSelf so that it is quick and easy to use and ultimately supports your needs.

As part of our research to examine the value of BrightSelf and technologies for the report of psychological wellbeing, you will be prompted infrequently over the course of 6 months to check and and check back using the application.

These prompts will appear as notifications on the front screen of your phone or as a banner appearing at the top.

For peer review only

These reports will be used entirely anonymously to inform our research and we would greatly appreciate if you could respond to the requests as accurately and timely as possible. At the end of this period you may then also be asked to complete a short questionnaire detailing your thoughts on the experience.

Through your feedback, and by examining, anonymously and securely, these responses, we hope to move towards improving the experience of all around the time of pregnancy.

The next section provides a quick guide to the installation and use of BrightSelf.

For peer review only - http://bmjopen.bmj.com/site/about/guidelines.xhtml

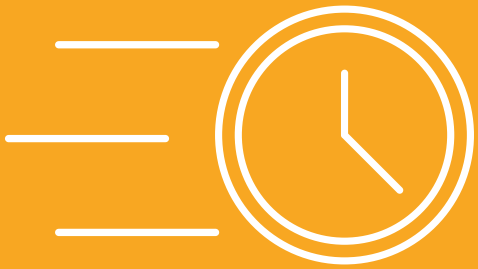

A Quick Guide

The Android Play Store Icon

The Apple App Store Icon

To use BrightSelf, the first step is to download the app to a phone by visiting the appropriate **app store**.

For peer review only

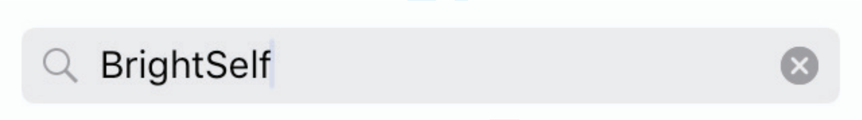

You can then search the store for BrightSelf. Look for the app icon and when you find it, choose to **install** it. It's free!

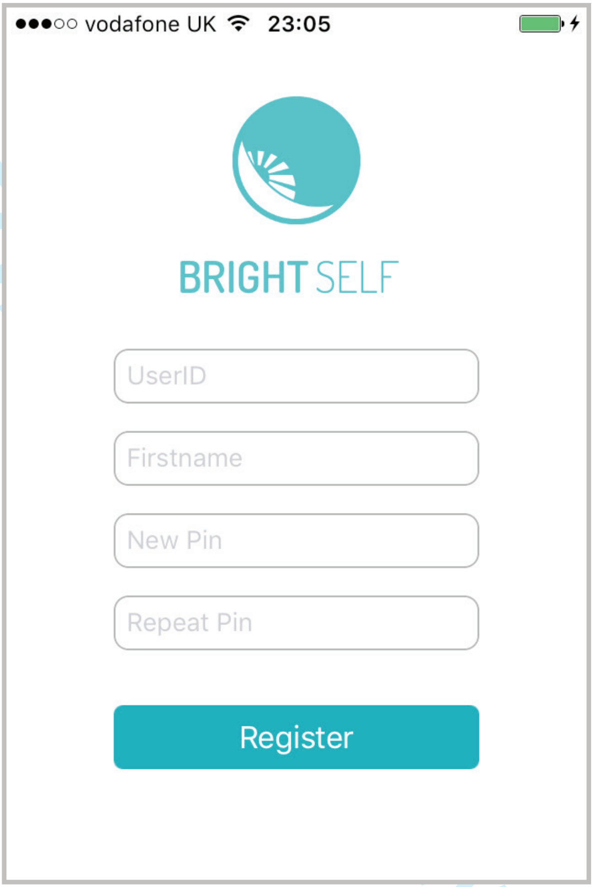

When you first open BrightSelf you will need to **register** a new user.

You may first be asked whether you wish to receive **push notifications** from BrightSelf.

If asked, please accept to do so!

For peer review only - http://bmjopen.bmj.com/site/about/guidelines.xhtml

In order to register, an internet connection (Wifi or mobile internet) is required. Once the device is connected, the user simply needs to enter the following information;

- **A 9-digit UserID** provided by the researchers. Please enter this carefully.
- The user's **Firstname** only (without numbers or spaces)
- **A 4-digit PIN** of the user's choosing which will allow her to login in the future. Please ensure to pick a pin which will not be forgotten!
- Enter this **PIN** a second time to ensure a match.

On clicking register, the system will then register the user for the study in several seconds. That's it, you're ready to explore BrightSelf!

For peer review only - http://bmjopen.bmj.com/site/about/guidelines.xhtml

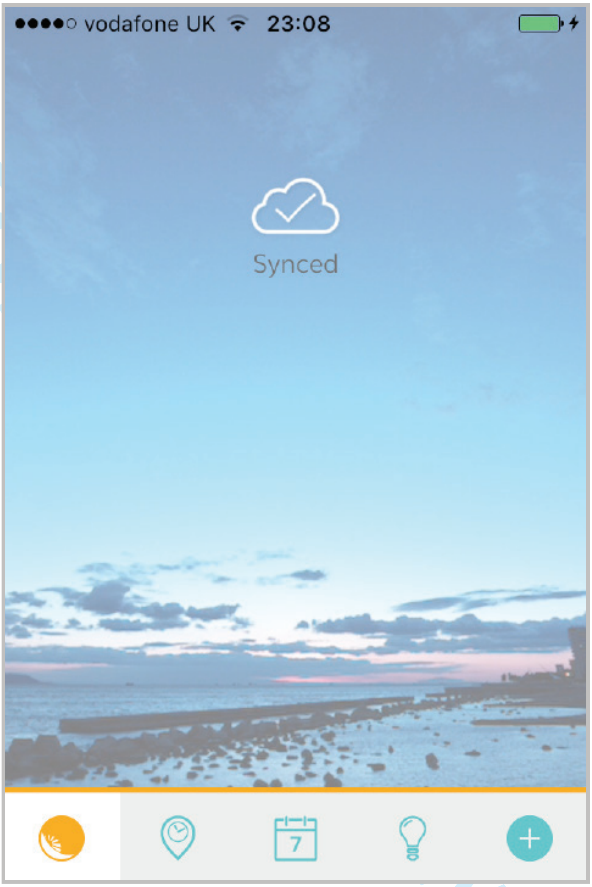

On the **home screen**, the syncing button shows whether your most recent reports have been uploaded, anonymously and securely, to our server for inclusion in our research. You can navigate through the app using the navigation bar located at the bottom of the screen.

For peer review only - http://bmjopen.bmj.com/site/about/guidelines.xhtml

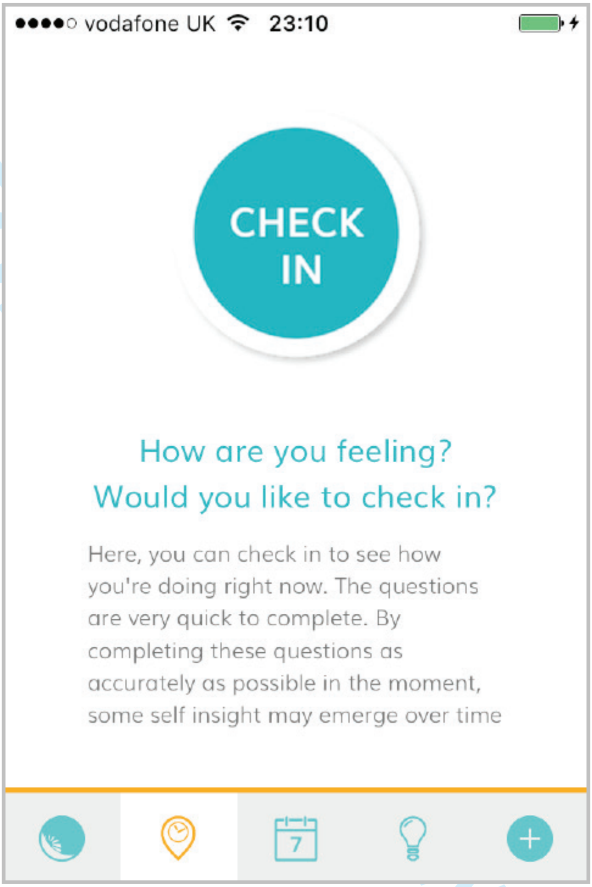

Checking in allows you to note how you are feeling right now by very quickly rating your mood, sleep, worry, enjoyment and energy.

For peer review only - http://bmjopen.bmj.com/site/about/guidelines.xhtml

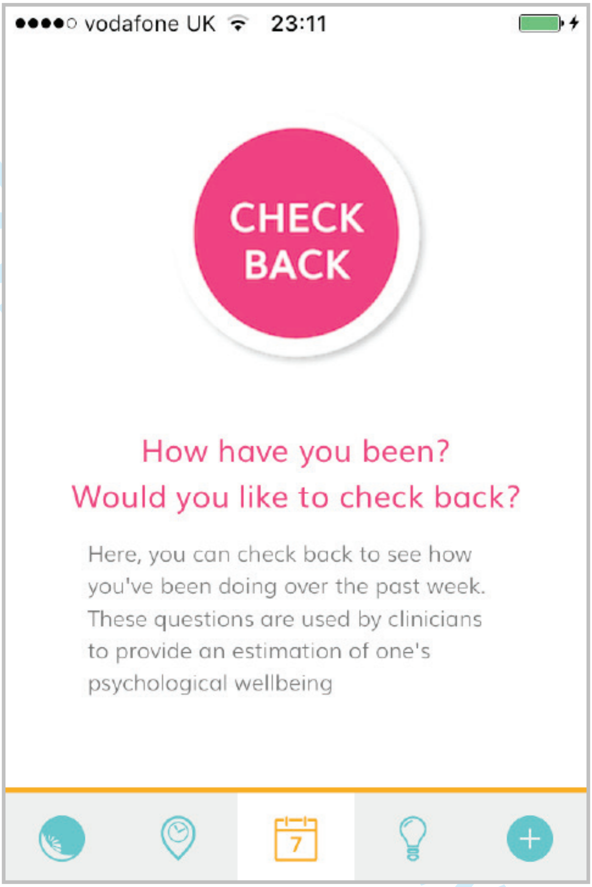

Checking back allows you to make note of your wellbeing over the past week by answering 10 questions which are normally used by clinicians to assess mood during pregnancy.

For peer review only - http://bmjopen.bmj.com/site/about/guidelines.xhtml

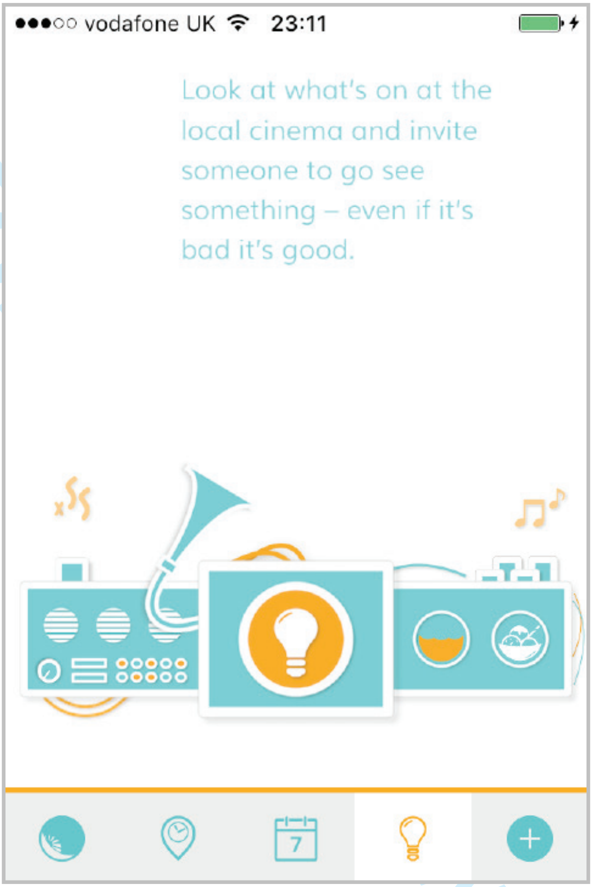

The **ideas machine** is there to provide that pick-me-up we all need from time to time. Remember however, that BrightSelf is not intended as a replacement for professional help. If at any point you feel that you need extra support, please do contact your GP or midwife.

For peer review only - http://bmjopen.bmj.com/site/about/guidelines.xhtml

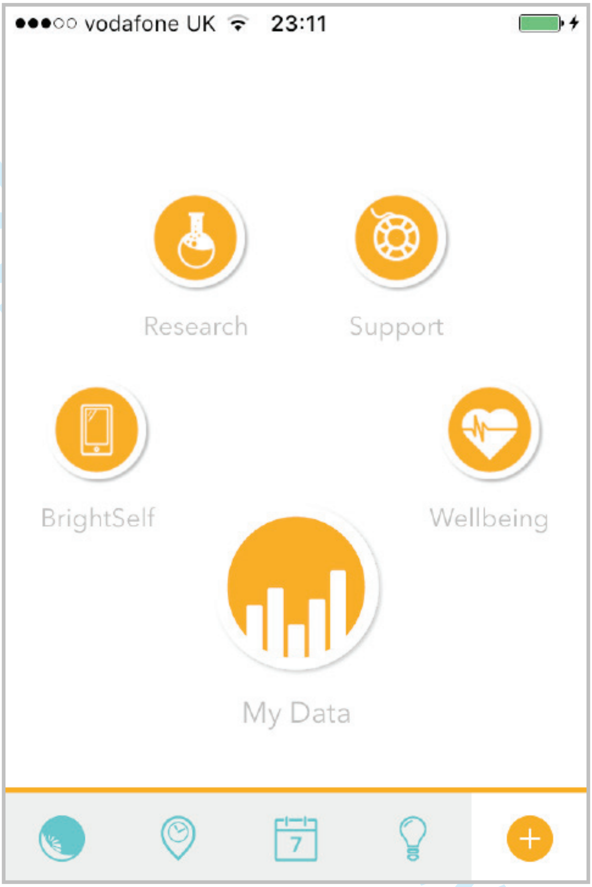

The **extras** section contains information about BrightSelf, wellbeing, and our research. Here you will also find additional sources of professional support should you need them.

For peer review only - http://bmjopen.bmj.com/site/about/guidelines.xhtml

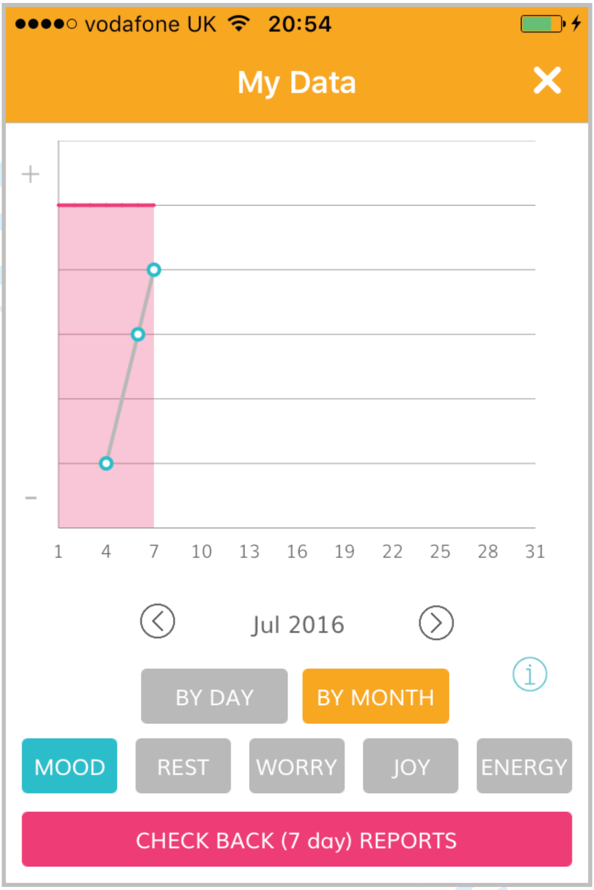

Under **My Data** you can interact with a plot of all your previous responses, allowing you to examine your reports over time, and empowering you to take control of your own wellbeing. Please note however, that these graphs do not represent any proven clinical value.

For peer review only - http://bmjopen.bmj.com/site/about/guidelines.xhtml

Finally, please note that you will receive infrequent **notifications** over the next 6 months which will request you to check in and check back. How these notifications appear will depend upon your device and some examples are shown below.

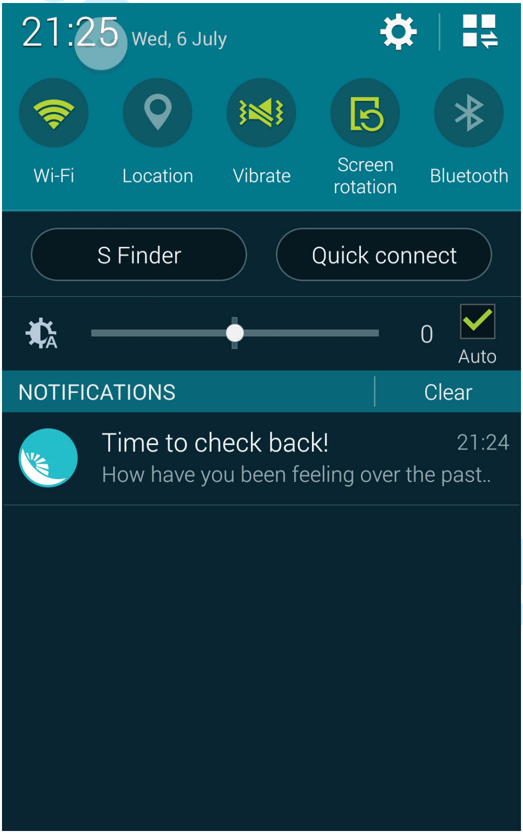

Android Notification

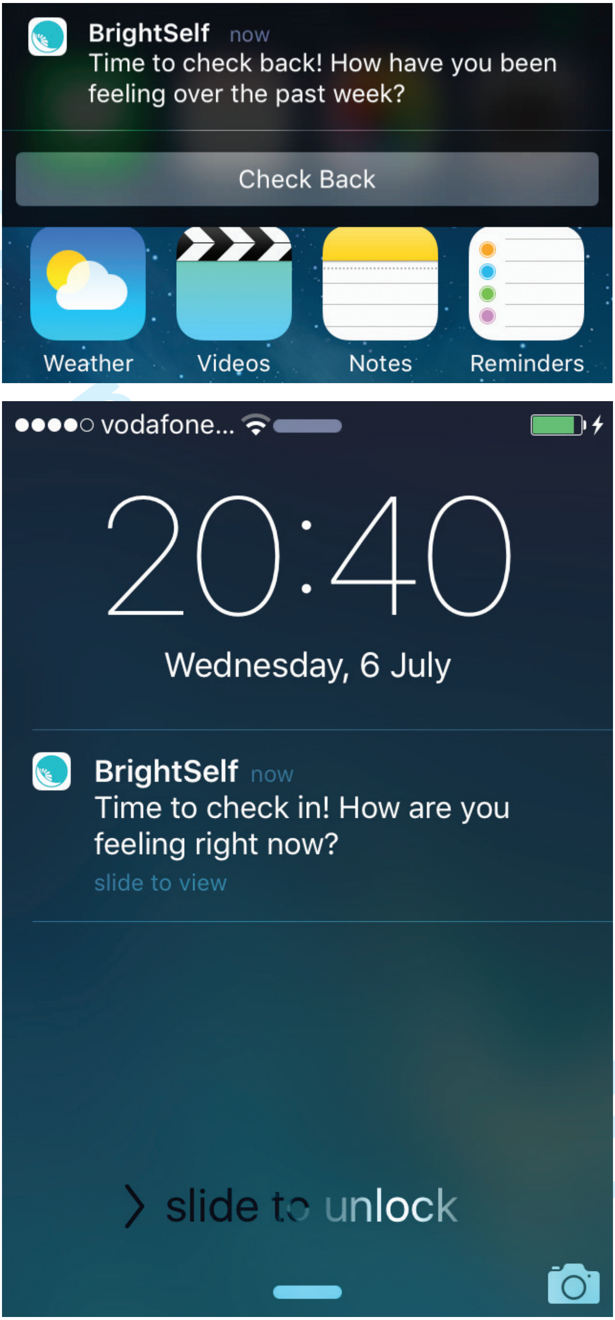

Apple Notification

Administrative Notes

Please note that the user's data is captured anonymously throughout the duration of the study and transferred to our servers using a secure connection.

No-one outside the research team will have access to the user's data. Any data gathered will be used for research purposes only.

This app is not intended to substitute the woman's care in any way but only to complement it. Please inform the user that if at any point she feels the need for extra support to contact her GP or midwife.

If any user's responses do indicate that she is struggling and might benefit from extra support, the system will alert the research team who will then contact the local research team to follow up.

For peer review only

Contact

At all times, our primary concern is with the user's wellbeing, privacy, and security.

Should you or any user have questions at any time please don't hesitate to contact us at **help@brightselfresearch.org**

Thank you once again for your participation and we hope you find participating in BrightSelf a valuable experience!

For peer review only

For peer review only

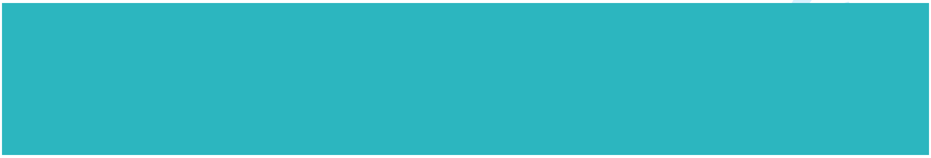

Appendix 3. Momentary and contextual questions

Momentary questions

1. I feel…
    a. Great
    b. Good
    c. Ok
    d. Not good
    e. Not good at all
2. How rested do I feel?
    a. Very rested
    b. Somewhat rested
    c. Neither rested nor tired
    d. Somewhat tired
    e. Very tired
3. How worried do I feel?
    a. Very worried
    b. Quite worried
    c. Somewhat worried
    d. A little worried
    e. Not at all worried
4. Am I enjoying myself?
    a. A lot
    b. Quite a lot
    c. Somewhat
    d. Not much
    e. Not at all
5. How energetic do I feel?
    a. Very energetic
    b. Fairly energetic
    c. Moderately energetic
    d. Not very energetic
    e. Not at all energetic

Contextual questions

1. Right now, I am…
    a. At home
    b. At work
    c. At the shops
    d. Outside
    e. At a Café or Restaurant
    f. In Transport
    g. At a Friend's or Family
    h. In a Clinic
    i. Other
2. Right now, I am…
    a. Relaxing

For peer review only

b. Working
c. Shopping
d. Looking After Others
e. Eating or Drinking
f. Travelling
g. Exercising
h. Waiting
i. None

For peer review only

Appendix 4. Post-study acceptance survey

Please rate your agreement with the following statements with respect to this system:

1. This app is easy to use
    - Strongly agree – Strongly disagree (7-point Likert Scale)
2. I learned to use this app quickly
    - Strongly agree – Strongly disagree (7-point Likert Scale)
3. I would recommend this app to a friend
    - Strongly agree – Strongly disagree (7-point Likert Scale)
4. I would repeat the experience of using this app
    - Strongly agree – Strongly disagree (7-point Likert Scale)
5. I found using this app an engaging experience
    - Strongly agree – Strongly disagree (7-point Likert Scale)
6. I found the assessments useful
    - Strongly agree – Strongly disagree (7-point Likert Scale)
7. I was asked to provide reports:
    - Much too infrequently
    - Too infrequently
    - The right amount
    - Too frequently
    - Much too frequently
8. The experience of using this app met my needs:
    - Strongly agree – Strongly disagree (7-point Likert Scale)
9. What were your motivations in using this app?
10. What did you like the most about the experience of using this app?
11. What did you like least about the experience of using this app?
12. How would you improve the experience of using this app?
13. Do you have any other comments that you would like to make?

To be answered by those participants allocated to the retrospective plus momentary assessment strategy:

14. I found it useful to compare reports made right now with those made over the past 7 days:
    - Strongly agree – Strongly disagree (7-point Likert Scale)

For peer review only

BMJ Open

A bespoke mobile application for the longitudinal assessment of depression and mood during pregnancy: protocol of a feasibility study

Journal:	*BMJ Open*
Manuscript ID	bmjopen-2016-014469.R1
Article Type:	Protocol
Date Submitted by the Author:	21-Dec-2016
Complete List of Authors:	Marcano-Belisario, Jose; Imperial College London, Global eHealth Unit, Department of Primary Care and Public Health<br>Doherty, Kevin; University of Dublin Trinity College, School of Computer Science and Statistics<br>O'Donoghue, John; Imperial College London, Global eHealth Unit, Department of Primary Care and Public Health<br>Ramchandani, Paul; University of Oxford, Psychiatry<br>Majeed, Azeem; Imperial College, Primary Care<br>Doherty, Gavin; University of Dublin Trinity College, School of Computer Science and Statistics<br>Morrison, Cecily; Imperial College London, Global eHealth Unit, Department of Primary Care and Public Health<br>Car, Josip; Imperial College London, Global eHealth Unit, Department of Primary Care and Public Health
**Primary Subject Heading**:	Health informatics
Secondary Subject Heading:	Mental health, Obstetrics and gynaecology, Public health
Keywords:	Depression & mood disorders < PSYCHIATRY, Ecological momentary assessment, mHealth, Antenatal care, Mobile apps

SCHOLARONE™
Manuscripts

A bespoke mobile application for the longitudinal assessment of depression and mood during pregnancy: protocol of a feasibility study

Corresponding author

José S Marcano-Belisario

Room 304, Reynolds Building, St Dunstan's Road, London, W6 8RP, UK

jose.marcano-belisario10@imperial.ac.uk

Telephone: +44 (0)20 7594 0771

List of authors

José S Marcano-Belisario

Global eHealth Unit, Department of Primary Care and Public Health, School of Public Health, Imperial College London, London, UK

Kevin Doherty

School of Computer Science and Statistics, Trinity College Dublin, Dublin, Ireland

John O'Donoghue

Global eHealth Unit, Department of Primary Care and Public Health, School of Public Health, Imperial College London, London, UK

Paul Ramchandani

The Centre for Mental Health, Department of Medicine, Imperial College London, London, UK

Azeem Majeed

Department of Primary Care and Public Health, School of Public Health, Imperial College London, London, UK

Gavin Doherty

School of Computer Science and Statistics, Trinity College Dublin, Dublin, Ireland

Cecily Morrison

Global eHealth Unit, Department of Primary Care and Public Health, School of Public Health, Imperial College London, London, UK

Josip Car

Global eHealth Unit, Department of Primary Care and Public Health, School of Public Health, Imperial College London, London, UK

For peer review only

Keywords

Perinatal depression

Mood

Ecological momentary assessment

Edinburgh Postnatal Depression Scale

Mobile phones

mHealth

Word count

4269 words

For peer review only

ABSTRACT

Introduction

Depression is a common mental health disorders during pregnancy, with important consequences for mothers and their children. Despite this, it goes undiagnosed and untreated in many women attending antenatal care. Smartphones could help support the prompt identification of antenatal depression in this setting. In addition, these devices enable the implementation of ecological momentary assessment techniques, which could be used to assess how mood is experienced during pregnancy. With this study, we will assess the feasibility of using a bespoke mobile application running on participants' own handsets for the longitudinal (6 months) monitoring of antenatal mood and screening of depression.

Methods and analysis

We will use a randomised controlled study design to compare two types of assessment strategies: retrospective + momentary (consisting of the Edinburgh Postnatal Depression Scale plus 5 momentary and 2 contextual questions), and retrospective (consisting of the Edinburgh Postnatal Depression Scale only). We will assess the impact that these strategies have on participant adherence to a pre-specified sampling protocol, drop-out rates and timeliness of data completion. We will evaluate differences in acceptance of the technology through a short quantitative survey and open ended questions. We will also assess the potential effect that momentary assessments could have on retrospective data. We will attempt to identify any patterns in app usage through the analysis of log data.

Ethics and dissemination

This study has been approved by the South East Coast – Surrey Research Ethics Committee. Our findings will be disseminated through academic peer-reviewed publications, conferences and discussion with peers.

Registration details

This study has been registered in ClinicalTrials.gov under the identifier NCT02516982.

For peer review only

STRENGTHS OF THIS STUDY

- This study will explore: (i) the role of mobile technology as a medium to address some of the practical barriers preventing depression screening in antenatal settings; (ii) how mood and depression are experienced throughout pregnancy (using momentary, experiential and ecological data); and (iii) two critical success factors for the successful deployment of mobile technology in pregnancy: user engagement and adherence to a proposed sampling protocol
- This study will provide baseline information regarding the appropriateness of a sampling protocol (in terms of its duration, intensity and frequency) for the monitoring of mood and screening of depression during the antenatal period
- The technology used in this study has been specifically designed and developed to fit within the clinical context and the local care pathways in which it will be deployed

LIMITATIONS OF THIS STUDY

- This study focuses on mood and antenatal depression. It does not consider other common mental health disorders that occur during pregnancy, or the presence of potential triggers or risk factors (for example, domestic violence)
- The mood-related momentary questions used in this study have not been validated
- This study relies on self-report measures. Research is being conducted to explore how smartphone and app usage data could be used to unobtrusively identify low mood and depression in the general population

For peer review only

INTRODUCTION

Antenatal depression is one of the most common, mental health disorders during pregnancy.[1-4] Point prevalence estimates vary between 7% and 12% (depending on the trimester), and period prevalence estimates suggest that as many as 12.7% of pregnant women could experience an episode of major depression.[3,5] Moreover, antenatal depression is associated with long-term adverse health outcomes in both mothers and their offspring. Pregnant women suffering from depression are more likely to engage in unhealthy practices (including poor diet, substance abuse, and failure to enroll in prenatal care), and are at increased risk of self-harm (or suicide) and postpartum depression.[1,6] Antenatal depression can also affect foetal development, and has been identified as an independent risk factor for a child's behavioural, cognitive and emotional development (including through adolescence).[1,7-9]

Research indicates that there is no difference in the prevalence or incidence of depression between pregnant and non-pregnant women.[10] However, the rate of diagnosis and treatment might be lower in pregnant women.[10] Approximately three-quarters of pregnant women meeting diagnostic criteria for depression (and anxiety) are not identified, and only 1 in 10 of those who require further treatment are able to access it.[11] Some of the barriers to the prompt diagnosis of this disorder include difficulties in differentiating depressive symptoms from the expected mood and somatic changes of pregnancy; stigma; lack of reassurance that mental health care is a normal part of antenatal care; characteristics of healthcare providers; configuration of health services; insufficient consultation time; and the cost-effectiveness of screening practices.[10-12]

As with other mental health conditions,[13-16] smartphones could help address some of the practical barriers and facilitate the screening and monitoring of depression throughout the antenatal period. The computational capabilities of these devices allow them to implement validated screening scales (usually retrospective self-reports) at any frequency and for any duration. Smartphones are also able to support the implementation of techniques for the collection of momentary, experiential and ecologically valid data. Being collected in real time, momentary data are less susceptible to many of the biases common to retrospective scales (e.g., recall bias), and are more sensitive to fluctuations over time.[17-19] The networking capabilities and wide availability (approximately 71% of UK adults)[20] of

For peer review only

smartphones, and the software development and distribution framework of mobile applications (apps), could help practitioners to circumvent some of the practical challenges associated with screening and clinical monitoring, and reduce the costs associated with data handling and management.

Altogether, these characteristics could contribute to making the screening and monitoring of antenatal depression more cost effective: an initial resource-intensive app development phase would be followed by a relatively low-cost, large scale distribution of the app onto patients own smartphones. Thereafter, regular depression assessments could take place remotely, at anytime and anywhere (further comprehensive clinical assessments and the provision of treatment being dependent on local referral and care pathways). Nonetheless, the feasibility of using smartphones for this purpose needs to be explored.

In this area of research, a key factor is patients' willingness to run screening or clinical monitoring apps on their personal handsets.[16] This could influence patient compliance with clinician-led data gathering (sampling) protocols, and thus affect data completeness. The latter refers to the minimum amount of information required by clinicians to inform their decisions, and is an important data quality dimension in healthcare.[21] Data completeness is also susceptible to the burden that the intensity of sampling protocols (both in terms of frequency and duration) can place on patients, as well as on the value that patients might derive from the data collected. Moreover, the impact that adding momentary assessments can have on retrospective data also needs to be explored, as this could lead to more efficient diagnostic and therapeutic decisions.

This study is part of a project aimed at understanding the role of mobile technology for the screening and assessment of antenatal depression and psychological wellbeing in the context of antenatal care pathways in the National Health Service (NHS) in the UK. A previous feasibility study assessed the feasibility of using iPads in the waiting area of antenatal clinics for implementing *The National Institute for Health and Care Excellence* (NICE) recommendations for the recognition of depression.[22] The present study will explore the feasibility of using a bespoke app to support the longitudinal and remote assessment of mood and depression screening throughout the antenatal period. We will evaluate issues of patient acceptance (namely, adherence to sampling protocols and drop out rates) by comparing two 6-month sampling protocols requiring either (i) monthly

For peer review only

retrospective *and* momentary assessments or (ii) monthly retrospective assessments.

For peer review only

METHODS AND ANALYSIS

study design

We will assess the feasibility of using a bespoke mobile application, called BrightSelf, running on participants' own smartphones to assess and monitor depression and mood during the antenatal period through a combination of retrospective assessments and ecological momentary assessments (EMA).

We will use a parallel, randomised controlled study design to assign our participants to one of two types of assessment strategies:

1. Retrospective plus momentary assessment: requiring the completion of (i) the Edinburgh Postnatal Depression Scale (EPDS), (ii) 5 momentary questions (assessing a participant's mood, sleep, worry, enjoyment and energy levels), and (iii) 2 contextual questions once a month for 6 months; or
2. Retrospective assessment: requiring the completion of the EPDS once a month for 6 months.

sample selection and recruitment

We will select our sample of participants from women attending antenatal clinics in general practices, community services, and secondary care NHS centres in England during their first 14 weeks of pregnancy. We have chosen this limit to ensure that most assessments will occur during pregnancy.

On the day of their antenatal appointment, each potential participant will be approached by a clinical studies officer (CSO) or a research midwife with appropriate good clinical practice (GCP) training, and will be provided with a participant information sheet. Potential participants expressing their interest in taking part in this study, will be assessed against our inclusion and exclusion criteria (Table 1).

Table 1. Participant inclusion and exclusion criteria

INCLUSION CRITERIA	EXCLUSION CRITERIA
Women who are 18 years old or older	Current diagnosis of depression or other mood disorder made by a health professional
Up to 14 weeks pregnant (assessed through a	Currently receiving treatment for depression or

For peer review only

dating ultrasound scan)	other mood disorder (whether it is talking therapies or pharmacological treatment)
Any parity	Recent personal history of depression or other mood disorder in the past 12 months
Attending antenatal clinics in participating NHS sites	Not comfortable reading and writing in English
Own smartphone (either an iPhone or any type of Android handset)	Not owning a smartphone, or owning an incompatible handset [i.e., Windows Phone, Blackberry, or Linux]

Potential participants meeting our inclusion criteria will have all the study details explained to them, and will be given the opportunity to ask as many questions about the study as they need. Potential participants will have a minimum of 24 hours to decide on participation; refusal to take part in this study will not have an impact on their legal rights, medical care or their relationship with care providers.

We will obtain written, informed consent from those potential participants who, after receiving all the relevant study information and having all their questions answered to their satisfaction, still wish to take part in this study.

After obtaining consent, participants will be asked to self-complete a baseline assessment using a tablet computer. This assessment will consist of a (i) socio-demographic survey, (ii) the Whooley questions, and (iii) the EPDS (Appendix 1). The last 2 instruments are recommended by the National Institute for Health and Care Excellence (NICE) to screen for depression during pregnancy.[23] Subsequently, the CSO or research midwife will guide participants through the process of downloading (from either the Apple AppStore or Google Play Store) and installing BrightSelf onto their own handsets. To this end, they will be able to use a recruiter booklet provided by the central research team (Appendix 2). In order to activate the app, participants will need to enter a 9-digit activation code, which will also be provided by the central research team.

interventions to be measured

Surveys

Non-validated, socio-demographic survey

For peer review only

We will administer an 11-question survey to collect information about participants' age group, ethnic background, marital status, employment status, level of education, smartphone and tablet computer ownership, obstetric history, and previous personal history of depression.

Whooley questions

The Whooley questions were developed as a case-finding instrument for depression in primary care.[24] This 2-question instrument assesses depressed mood and anhedonia that have been present during the past month. Respondents are required to answer *Yes* or *No* to each question.

Edinburgh Postnatal Depression Scale

The EPDS is a 10-item self-administered survey that was originally developed to screen for postpartum depression.[25] Since then it has been validated for use in the perinatal period and for use in community and clinical settings. This instrument assesses feelings of guilt, sleep disturbance, anhedonia and suicidal ideation that have been present during the past 7 days. Each question is scored on a 4-point scale ranging from 0 to 3 points. An overall score is generated from the sum of these responses.

Although there is variability in the diagnostic accuracy of different EPDS scores, the following thresholds are commonly used: 10 points for possible depression, 13 for probable depression, and 15 for antenatal depression.[26] In addition, special attention should be paid to item 10, as it deals with suicidal thoughts. Based on these scores, a clinician would be prompted to refer a woman to a mental health professional.

The EPDS is a valid and reliable tool for identifying women who are at risk of depression, both during pregnancy and postpartum. This instrument is also sensitive to changes in the severity of depression over time.[25] The EPDS can be reproduced without further permission provided that the original source of the scale is cited in each reproduced copy.

Non-validated, momentary mood questions

We will administer 5 momentary questions to assess participants' mood, sleep, worry, enjoyment and energy (Appendix 3). These questions are based on the work of a research fellow at the Collaboration for Leadership and Applied Health Research and Care (CLAHRC)

For peer review only

for the East of England.[27] Each question will be mapped onto 5-point pictorial scales, ranging from 1 (low) to 5 points (high). For the purpose of this feasibility study, we will not perform any overall score calculation or attempt any validation of these questions.

Non-validated, contextual questions

Two contextual questions will complement the momentary mood questions (Appendix 3). They will assess (i) participants' location and (ii) the activity in which they were engaged at the time they were required to complete the 5 momentary mood questions.

Non-validated, post-study acceptance survey

We will administer 13 questions (to participants completing retrospective assessments) or 14 questions (to participants completing retrospective plus momentary assessments) at the end of the 6-month participation period (Appendix 4). The purpose of these questions is to assess the acceptability to participants of BrightSelf in the context of their antenatal care, and to gather information about their experience of using it. These questions were derived from the USE questionnaire[28] (which focuses on usability) and also include a question concerning the desire to continue use (as used to assess engagement[29]), as well as questions regarding the experience of use and self-report. We will administer them as a web survey through Snap® survey software[30] by sending participants a link via email or SMS.

Mobile System

BrightSelf is a mobile system for the collection of self-reports, both retrospective and momentary, during pregnancy. This system is not a diagnostic tool, and is not intended to replace the role of clinicians within antenatal or mental health care pathways.

This system elicits retrospective reports through the EPDS, and momentary reports according to the 5 constructs of mood, sleep, worry, enjoyment and energy. In addition, the system can suggest brief activities that users can perform to lift their mood; additional information about the system and the feasibility study; additional resources should they need immediate help; and a visualisation of past self-reports. We will emphasise to participants that BrightSelf is not a clinical tool, and that if they feel they need immediate help at any point during the study they should contact their GP.

For peer review only

BrightSelf consists of mobile applications for Android and iOS operating systems, a backend for the storage and management of data, and a website which supports the monitoring of data.

A complete description of BrightSelf is the aim of another publication. Here we describe the two features that are most relevant to this feasibility study, namely, the collection of retrospective and momentary self-reports.

Check Back

This feature will enable the administration of the EPDS. It consists of two introductory screens informing participants of the retrospective nature of this scale, and its intended applications. These screens will be followed by the 10 EPDS questions, presented one question per screen, using radio buttons to capture participants' responses (Figure 1).

Check In

This feature will enable the administration of the 5 momentary questions and the 2 contextual questions. The first 5 questions will be presented using a 5-point pictorial scale with temporary supporting text (see Figure 2 for an example). The last 2 questions will use radio buttons to capture participants' responses.

Retrospective versus momentary assessments

Participants will be asked to complete monthly assessments at irregular intervals for 6 months. Whenever an assessment is due (as per the sampling protocol), a notification will be displayed on a participant's handset. There will be two types of notifications: one for the *Check Back* feature and one for the *Check In* feature of BrightSelf. The visual appearance of the notifications will depend on the operating system and on the model of the participant's handset. The text of the notifications however, will remain constant across participants for each type of notification.

Participants will be able to respond (and thus complete the corresponding assessments) or to dismiss the notifications. In addition, participants will be able to leave notifications unanswered. If participants do not complete an assessment in response to a notification, they will not receive reminders or follow-up notifications. In these instances, the non-response will be recorded in our database and will be coded as such for the purposes of our

For peer review only

data analysis.

Outside assessment periods, participants will be able to use BrightSelf in any way they find convenient and there will be no restrictions on the number of assessments they can complete on a single day. However, we will be able to distinguish between assessments completed in response to a notification and those completed spontaneously.At baseline, participants will be randomly allocated to one of two types of assessment strategies:

Retrospective plus Momentary Assessments

In this experimental manipulation, participants will be asked to complete a combination of retrospective assessments and momentary assessments. A retrospective assessment will be defined as a single administration of the EPDS. A momentary assessment will be defined as the 5 momentary questions plus the 2 contextual questions.

Participants will be required to complete assessments for a total of 6 months, and each assessment period will consist of 6 consecutive days. The 6-month participation period will be calculated from the day on which the app is activated (i.e., when participants enter their activation code and activate their account), and it will be divided into six intervals. All participants will receive the first notification 2 days after activating BrightSelf (i.e., first assessment), while the use of the app is fresh within their minds and to give an idea of what to expect during the study. Subsequent assessment periods will be a random selection of 6 consecutive days within the 21 to 35 days following the end of the previous assessment period (in order to avoid 12 days of continuous assessments).

The assessment period will be structured as follows:

- Day 1: one retrospective assessment at any random time between 17:00 and 21:00;
- Day 2 to 5: 3 momentary assessments per day, displayed at random times within each of the following intervals: 09:00 – 12:00; 13:00 – 16:00; and 17:00 – 20:00; and
- Day 6: one retrospective assessment at any random time between 17:00 and 21:00.

At the end of the 6-month period, participants will be sent a link via short messaging service (SMS) or by email to complete the non-validated, post-study acceptance survey.

Retrospective Assessments

In this experimental manipulation, participants will be asked to complete retrospective

For peer review only

assessments only. A retrospective assessment will be defined as a single administration of the EPDS.

Participants will be required to complete assessments for a total of 6 months, and each assessment period will consist of one day. The 6-month participation period will be calculated from the day on which the app is activated (i.e., when participants enter their activation code and activate their account), and it will be divided into six intervals. Again, all participants will receive the first notification 2 days after activating BrightSelf (i.e., first assessment). Subsequent assessments will take place on a random day within the 21 to 35 days following the end of the previous assessment period (in order to avoid two consecutive EPDS assessments).

On each assessment day, participants will receive a notification to complete the EPDS at a random time between 17:00 and 21:00.

At the end of the 6-month period, participants will be sent a link via SMS or by email to complete the non-validated, post-study acceptance survey.

See Figure 3 for an illustration of both sampling protocols.

Duty of care

Regardless of participants' allocation, the central research team will be alerted if any of a series of pre-specified conditions are met (Table 2). These alerts will be colour-coded according to their severity level. In order to determine the severity level, we have chosen commonly used EPDS scoring thresholds (i.e., 10 – 12 points for possible depression, and 13 points or more for probable depression). We acknowledge the limitations of this approach as the diagnostic accuracy of the EPDS can vary depending on the setting and the population in which it is administered.

Table 2. Criteria for an alert to be sent to the research team

TYPE OF ALERT	CRITERIA
Yellow [Mild]	Completing more than one EPDS assessment on the same day, with an overall EPDS score of 9 points or less and a score of 0 on question 10 of the EPDS

For peer review only

Orange [Moderate]	Overall EPDS score between 10 and 12 points, with a score of 0 on question 10 of the EPDS
Red [Severe]	Overall EPDS score of 13 points or more<br><br>1 or more points on question 10 of the EPDS, regardless of the overall EPDS score

Before starting the study, the central research team will agree a list of designated contacts with the relevant clinical care teams. These contacts will include clinicians who are available during normal working hours, as well as those on duty outside working hours (including weekends and bank holidays). If a red or orange alert is generated, the study coordinator will contact the designated member of the clinical care team by phone and email within 24 hours of receiving the alert. The clinical care team will then follow up these alerts directly with the participants.

randomisation

We will use block randomisation procedures (with blocks of 4) to allocate participants to one of the two experimental arms. Random numbers will be generated using Stata 14.[31] Each consecutive number will be embedded within the 9-digit activation codes that will be distributed to each participating NHS sites. The full activation codes will be generated in the same order in which the random numbers were generated. Each NHS site will receive a list of activation codes on a first-come-first-served basis. Members of the local research teams will not be informed of the random sequence generation and will not be able to identify which number determines participant allocation. They will be required to use activation codes sequentially, as participants are recruited. Activation codes referring to the *Retrospective plus momentary assessment* condition will activate a version of BrightSelf in which both the *Check Back* and the *Check In* features are active. Activation codes referring to the *Retrospective assessment* condition will activate a version of BrightSelf in which only the *Check Back* feature is active.

outcomes

Adherence to sampling protocols

For peer review only

We will calculate the number of participants who complete 100% of the expected assessments (as per the study protocol) as a proportion of the total number of participants who were randomised into the study. We will sub-divide this outcome by using the total number of participants who complete the 6-month participation period as the denominator.

Drop-out rates

We will calculate the number of participants who complete the 6-month participation period as a proportion of the total number of participants who were randomised into the study.

Usage patterns

We will assess participants' usage pattern of BrightSelf by analysing log data (additional variables capturing app usage). These will include the number of additional voluntary self-reports, time spent using the app, number of interactions with other sections of the app, time taken to complete the self-assessments, and other ancillary usage data.

Acceptance

We will calculate participants' ratings and responses to the post-study acceptance survey. In addition, we will conduct thematic analysis of participants' answers to the open-ended questions of this survey using Atlas.ti 8.[32]

Timeliness of data completion

We will calculate the number of assessments that were completed in response to a notification, as a proportion of the total number of expected assessments. We will sub-divide this outcome by using the total number of completed assessments as the denominator. We will consider that an assessment has been completed in response to a notification if it takes place within the interval corresponding to that notification (i.e., before the next notification is delivered). This broad interval may raise concerns regarding the ecological validity of the report, which we will explore in our statistical analysis.

sample size calculations

We have chosen to relate the proposed sample size to the 95% confidence interval for the adherence rate, as recommended by the Research Design Service in London. Therefore, we would need 96 participants in each arm with a 95% confidence level and a confidence

For peer review only

interval of 10. This translates into a total sample of 192 (i.e., 96 participants in each experimental group). For this reason, we will aim to recruit at least 250 participants, to account for drop-outs and potential miscarriages.

data analysis plan

Descriptive statistics

We will report the number of potential participants who were eligible and refused to take part in the study. Where possible, we will report the reasons for refusing to participate.

For each experimental group, we will report the following information:

- Demographic characteristics (as captured by the non-validated, socio-demographic survey);
- Proportion of participants answering Yes to any of the Whooley questions during the baseline assessment;
- Proportion of participants scoring at each interval of the EPDS: between 0 and 9 points; between 10 and 12 points; and 13 points or above during the baseline assessment; and
- Proportion of participants scoring 1 or more points on question 10 of the EPDS during the baseline assessment

Inferential statistics

We will compare the *Retrospective and Momentary assessment* and the *Retrospective assessment* experimental groups for differences in adherence rates, drop-out rates and timeliness of data completion. For this we will use a t-test or the non-parametric equivalent.

We will compare acceptance between the two experimental manipulations by assessing differences in participants' rankings to those questions on a 7-point Likert scale of the post-study acceptance survey. We will also conduct a thematic analysis of the open-ended questions of this survey.

In relation to the momentary assessments, we will examine the distribution of delays between the time of notification and time of report in order to assess the effect of this delay on the ecological validity of reports.

We will analyse usage patterns through regression modelling of log data. We will analyse

For peer review only

participants momentary and retrospective assessments through time-based analyses or multi-level modelling.[33,34] We will attempt to compare if the momentary assessments had any effect on the retrospective assessments by comparing the EPDS responses given on Day 1 to those given on Day 6.

timeline

We expect participant recruitment to start in January 2017, and the last follow up to take place in August/September 2017 (assuming a recruitment period of 2 months and a half).

For peer review only

CONCLUSION

This study addresses an important area of unmet clinical need, with direct and indirect consequences for mothers, children, their families, health systems and society. This study will contribute to the growing body of evidence concerning the role of mobile technologies for the support of mental health. Similarly, it will generate baseline information concerning the acceptability of an EMA sampling protocol (in term of its duration, frequency and intensity) to women attending antenatal care. This study will also evaluate participant engagement with the technology and their adherence to a pre-specified sampling protocol; both of which influence the completeness of the data needed by clinicians to inform their decisions. In addition, this study will identify some of the implementation issues that might arise when we attempt to deploy mobile technologies in clinical settings, which could affect their successful adoption.

In this work, we have maintained a focus on antenatal depression. Mental health during pregnancy however, is more complex. Disorders such as anxiety and post-traumatic stress disorder are also amongst the most common disorders during this period. Moreover, pregnant women who are exposed to certain risk factors or triggers (e.g., domestic violence, social isolation, minority groups, and low-income) are at increased risk of suffering from any of these mental health problems. Although we have not been able to focus on all these issues, we believe that the findings from this feasibility study will produce important lessons that could enable us to design and develop similar technologies and appropriate strategies. We aim to recruit participants from diverse backgrounds, and across multiple settings and geographical areas within England, and to focus on how such technologies can be embedded within existing antenatal care pathways, in order to support the existing patient-midwife relationship.

The findings from this and from a previous feasibility study[19] will inform a larger trial evaluating the integration of mobile technologies into routine antenatal pathways, and their potential impact on clinical outcomes.

For peer review only

ETHICS AND DISSEMINATION

This study has been reviewed and approved by the National Research Ethics Service (NRES) Committee South East Coast – Surrey on 15 April 2016 as a notice of substantial amendment to the original submission (09 July 2015) under the Research Ethics Committee (REC) reference 15/LO/0977.

This study is being sponsored by Imperial College London under the reference number 15IC2687 and has been included in the UK Clinical Research Network (CRN) Study Portfolio under the Central Portfolio Management System (CPMS) number 19280.

Lastly, this study protocol has been registered in ClinicalTrials.gov under the identifier NCT02516982 (as required by the REC).

The findings of this study will be disseminated through academic peer-reviewed publications, poster presentations and abstracts at academic and professional conferences, discussion with peers, and social media. The findings of this study will also inform the PhD theses of Jose Marcano Belisario and Kevin Doherty.

For peer review only

REFERENCES

1 Stewart DE. Depression during pregnancy. N Engl J Med 2011;365:1605-11.

2 Mental Health Foundation. Fundamental Facts About Mental Health 2015. London: Mental Health Foundation 2015.

3 Gavin NI, Gaynes BN, Lohr KN, et al. Perinatal depression: a systematic review of prevalence and incidence. Obstet Gynecol 2005;106(5 Pt 1):1071-83 doi:10.1097/01.AOG.0000183597.31630.db.

4 Lee AM, Lam SK, Sze Mum Lau SM, et al. Prevalence, course, and risk factors for antenatal anxiety and depression. Obstet Gynecol 2007;110(5):1102-12 doi:10.1097/01.AOG.0000287065.59491.70

5 Bennett HA, Einarson A, Taddio A, et al. Prevalence of depression during pregnancy: systematic review. Obstet Gynecol 2004;103(4):698-709 doi:10.1097/01.AOG.0000116689.75396.5f.

6 Heron J, O'Connor TG, Evans J, et al. The course of anxiety and depression through pregnancy and the postpartum in a community sample. J Affect Disord 2004;80(1):65-73 doi:10.1016/j.jad.2003.08.004

7 Kinsella MT, Monk C. Impact of Maternal Stress, Depression & Anxiety on Fetal Neurobehavioral Development. Clin Obstet Gynecol 2009;52(3):425-440 doi:10.1097/GRF.0b013e3181b52df1.

8 Pearson RM, Evans J, Kounali D, et al. Maternal Depression During Pregnancy and the Postnatal Period: Risks and Possible Mechanisms for Offspring Depression at Age 18 Years. JAMA Psychiatry 2013;70(12):1312-9 doi:10.1001/jamapsychiatry.2013.2163.

9 Braithwaite EC, Murphy SE, Ramchandani PG. Effects of prenatal depressive symptoms on maternal and infant cortisol reactivity. Arch Womens Ment Health 2016;19:581-590 doi:10.1007/s00737-016-0611-y

10 Howard LM, Molyneaux E, Dennis CL, et al. Non-psychotic mental disorders in the perinatal period. Lancet 2014;384(9956):1775-88 doi: 10.1016/S0140-6736(14)61276-9 [published Online First: 14 November 2014].

For peer review only

11 Kingston D, Austin MP, Heaman M, et al. Barriers and facilitators of mental health screening in pregnancy. J Affect Disord 2015;186:350-7 doi:10.1016/j.jad.2015.06.029 [published Online First: 29 July 2015].

12 Goodman JH. Women's attitudes, preferences, and perceived barriers to treatment for perinatal depression. Birth 2009;36(1):60-9 doi:10.1111/j.1523-536X.2008.00296.x.

13 BinDhim NF, Alanazi EM, Aljadhey H, et al. Does a Mobile Phone Depression-Screening App Motivate Mobile Phone Users With High Depressive Symptoms to Seek a Health Care Professional's Help? J Med Internet Res 2016;18(6):e156 doi:10.2196/jmir.5726.

14 BinDhim NF, Shaman AM, Trevena L, et al. Depression screening via a smartphone app: cross-country user characteristics and feasibility. J Am Med Inform Assoc 2015;22(1):29-34 doi:10.1136/amiajnl-2014-002840 [published Online First: 17 October 2014]

15 Woo YS, Bahk WM, Hong J, et al. Use of a smartphone application to screen for bipolar sprectrum disorder in a community sample. Health Informatics J 2016;22(3):779-88 doi:10.1177/1460458215589601 [published Online First: 17 July 2015].

16 Torous J, Chan SR, Yee-Marie Tan S, et al. Patient Smartphone Ownership and Interest in Mobile Apps to Monitor Symptoms of Mental Health Conditions: A Survey in Four Geographically Distinct Psychiatric Clinics. JMIR Mental Health 2014;1(1):e5 doi:10.2196/mental.4004.

17 Moskowitz DS, Young SN. Ecological momentary assessment: what it is and why it is a method of the future in clinical psychopharmacology. J Psychiatry Neurosci 2006;31(1):13-
18 Shiffman S, Stone AA, Hufford MR. Ecological momentary assessment. Annu Rev Clin Psychol 2008;4:1-32.

19 Asselbergs J, Ruwaard J, Ejdys M et al. Mobile Phone-Based Unobtrusive Ecological Momentary Assessment of Day-to-Day Mood: An Explorative Study. J Med Internet Res 2016;18(3):e72 doi:10.2196/jmir.5505.

20 Ofcom. Facts & Figures. http://media.ofcom.org.uk/facts/ [date accessed: 30 August 2016].

21 Marcano-Belisario JS, Jamsek J, Huckvale K, et al. Comparison of self-administered survey questionnaire responses collected using mobile apps versus other methods. Cochrane

For peer review only

Database Syst Rev 2015;27(7):MR000042 doi:10.1002/14651858.MR000042.pub2 [published Online First: 27 July 2015].

22 Marcano-Belisario JS, Gupta AK, O'Donoghue J, et al. Tablet computers for implementing NICE antenatal mental health guidelines: protocol of a feasibility study. BMJ Open 2016;6:e009930 doi:10.1136/bmjopen-2015-009930 [published Online First: 22 January 2016].

23 National Collaborating Centre for Mental Health. Antenatal and postnatal mental health: clinical management and service guidance—updated edition. Leicester and London: The British Psychological Society and The Royal College of Psychiatrists, 2014.

24 Whooley MA, Avins AL, Miranda J, et al. Case-finding instruments for depression – Two questions are as good as many. J Gen Intern Med 1997;12(7):439-45.

25 Cox JL, Holden JM, Sagovsky R. Detection of Postnatal Depression - Development of the 10-Item Edinburgh Postnatal Depression Scale. Br J Psychiatry 1987;150:782-86.

26 Gibson J, McKenzie-McHarg K, Shakespeare J, et al. A systematic review of studies validating the Edinburgh Postnatal Depression Scale in antepartum and postpartum women. Acta Psychiatr Scand 2009;119(5):350-64 doi:10.1111/j.1600-0447.2009.01363.x [published Online First: 2 March 2009].

27 Blake F. Mumshine – The App: The design of a micro on-line intervention for postnatal women to improve mood and well-being using an app for the smart phone. Presented at: CLAHRC EAST OF ENGLAND FELLOWS SHOWCASE EVENT. 4 December 2014.

28 Lund AM. Measuring usability with the USE questionnaire. Usability Interface 2001;8(2):3-6.

29 Schoenau-Fog H. (2011). Hooked!–evaluating engagement as continuation desire in interactive narratives. In Interactive storytelling (pp. 219-230). Springer: Berlin, Heidelberg

30 Snap® Surveys Limited. Powerful and flexible software. [Online] Available at: http://www.snapsurveys.com/survey-software/ [accessed 14 December 2016]

31 Stata. Stata Statistical Software [Program]. Version 14, 2015.

32 Atlas.ti. Atlas.ti Qualitative Data Analysis [Program]. Version 8, 2016.

For peer review only

33 Schwartz JE, Stone AA. Strategies for analyzing ecological momentary assessment data. Health Psychol 1998;17(1):6-16.

34 Shiffman S. Conceptualizing Analyses of Ecological Momentary Assessment Data. Nicotine Tob Res 2014;16(Suppl 2):S76-S87 doi:10.1093/ntr/ntt195 [published First Online: 9 December 2013].

For peer review only

AUTHORS' CONTRIBUTIONS

José S Marcano-Belisario (JMB) developed the study protocol in collaboration with Kevin Doherty (KD). JMB was in charge of obtaining ethics and governance approvals, and has been approaching potential participant recruitment centres. KD was in charge of software development. JMB drafted this manuscript. Cecily Morrison (CM), Gavin Doherty (GD), Paul Ramchandani (PR), John O'Donoghue (JOD), Azeem Majeed (AM), and Josip Car (JC) guided the development of the study protocol. CM, JOD and JC have supervised JMB's work. GD has supervised KD's work. All the authors reviewed and approved this manuscript.

ACKNOWLEDGEMENTS

We would like to thank Prof Susan Ayers and Alexandra Thornton at City University London, Joanna Girling and Marie O'Connell at West Middlesex Hospital, Tara Pauley and Charlotte Clayton at Hinchingbrooke Hospital, Antoinette McNulty and Gemma Loebenberg at the Northwest London CRN, and Dr Fiona Blake for their feedback on early versions of BrightSelf and the study protocol, and for their help with our patient and public involvement initiatives. Thank you to Marguerite Barry at Trinity College Dublin for running some of the patient and public involvement sessions with us.

FUNDING STATEMENT

This work is being supported by a National Institute for Health Research (NIHR) Imperial Biomedical Research Centre (BRC) award through the Population Health Theme, and by the ADAPT Centre through Trinity College Dublin.

COMPETING INTERESTS STATEMENTS

The authors declare that they do not have any competing interests.

For peer review only

LIST OF FIGURES

Figure 1. BrightSelf – Check Back (screenshot)

Figure 2. BrightSelf – Check In (screenshot)

Figure 3. Sampling protocols for the retrospective plus momentary assessments and for the retrospective assessments

For peer review only

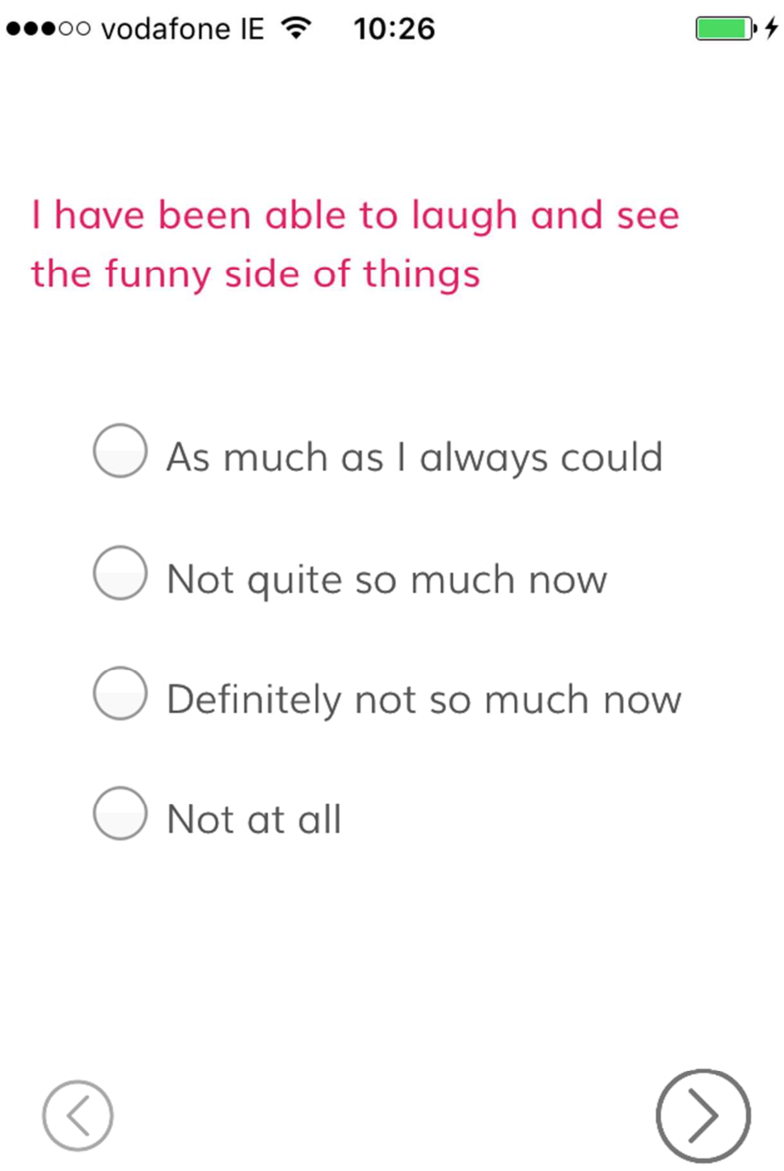

Figure 1. BrightSelf - Check Back (screenshot)

54x81mm (300 x 300 DPI)

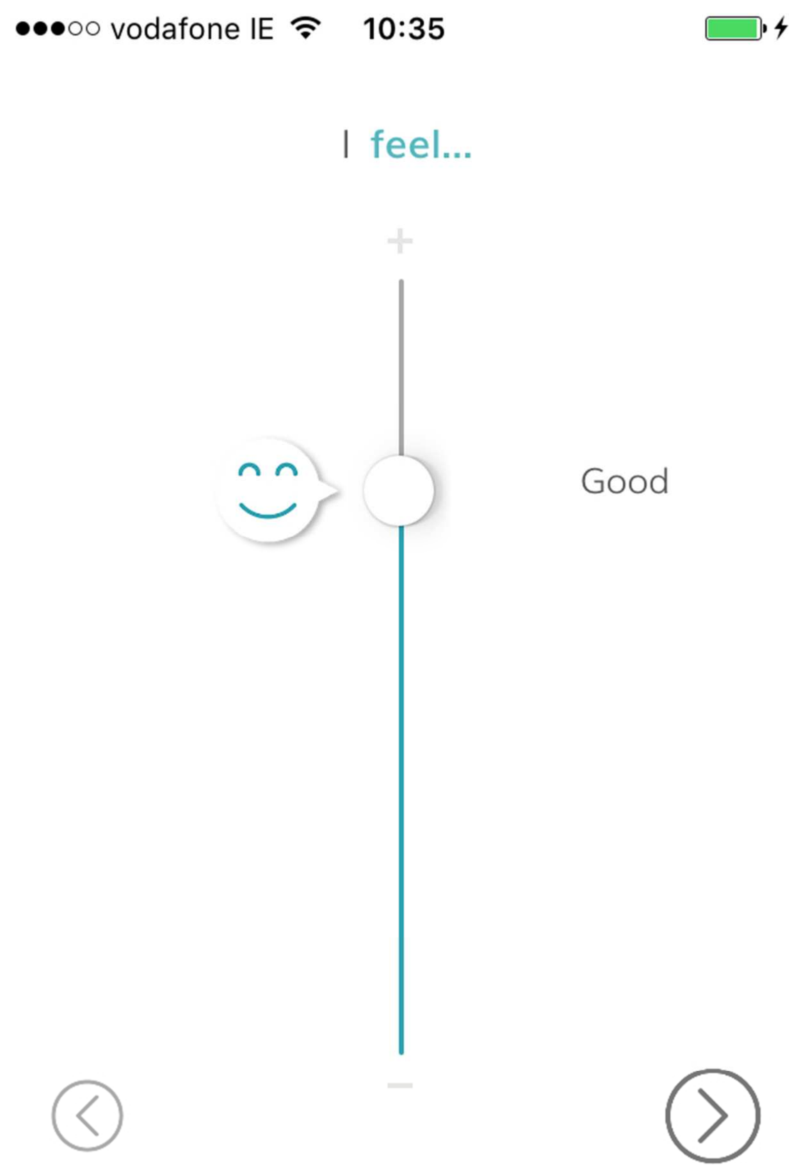

Figure 2. BrightSelf - Check In (screenshot)

54x81mm (300 x 300 DPI)

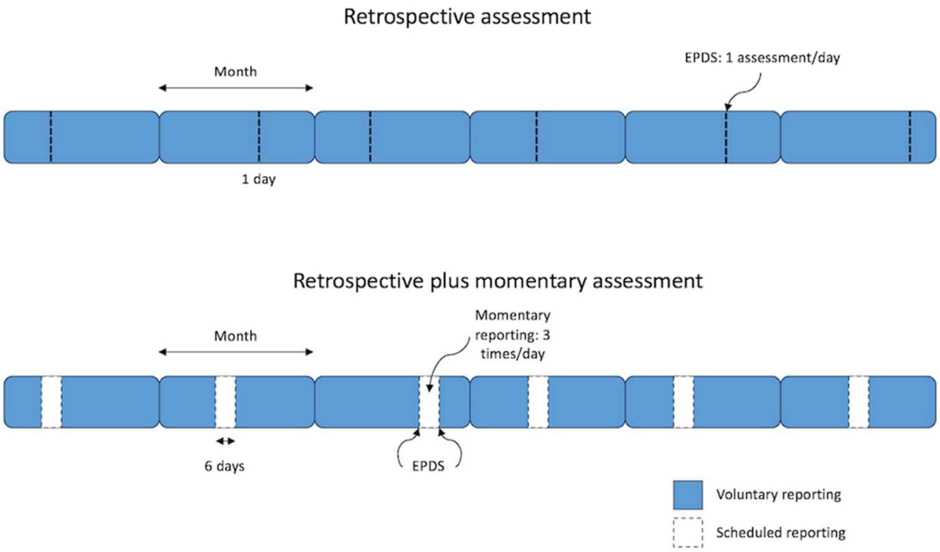

Figure 3. Sampling protocols for the retrospective plus momentary assessments and for the retrospective assessments

84x52mm (300 x 300 DPI)

For peer review only

Appendix 1. Baseline assessments

Socio-demographic survey

- What is your age group?
  - 18 to 22 years
  - 23 to 27 years
  - 28 to 32 years
  - 33 to 37 years
  - 38 years or older
- How would you describe your race or ethnicity?
  - White
    - British
    - Irish
    - Other
  - Mixed
    - White and Black Caribbean
    - White and Black African
    - White and Asian
    - Other
  - Asian or Asian British
    - Indian
    - Pakistani
    - Bangladeshi
    - Other
  - Black or Black British
    - Caribbean
    - African
    - Other
  - Chinese
  - Other
  - Prefer not to say
- What is your relationship status?
  - Single
  - Married/In a civil partnership
  - Living with partner
  - Divorced/Civil partnership that has been dissolved
  - Widowed
  - Separated
  - Prefer not to say
- What is your employment status?
  - Employed, full-time
  - Employed, part-time
  - Self-employed
  - Not employed, looking for work
  - Not employed, not looking for work
  - Disability/Not able to work

For peer review only

- What is your highest level of education?
  - Postgraduate – PhD/Doctorate
  - Postgraduate – Masters degree
  - University or college degree
  - University or college qualification below degree level
  - A Levels or equivalent
  - GCSE or equivalent
  - Other
  - None of these
- Do you own a smartphone (for example, an iPhone or Samsung phone)?
  - Yes
  - No
- Do you own a tablet computer (for example, an iPad)?
  - Yes
  - No
- How many times have you been pregnant (please count your current pregnancy)?
  - 1
  - 2
  - 3
  - 4 or more
- How many children have you given birth to?
  - 0
  - 1
  - 2
  - 3 or more
- When is your baby due? (please indicate month and year)
- Have you ever been diagnosed with depression?
  - Yes
  - No

Whooley questions

- Over the past month, have you been bothered by feeling down, depressed or hopeless?
  - Yes
  - No
- Over the past month, have you been bothered by having little interest or pleasure in doing things?
  - Yes
  - No

Edinburgh Postnatal Depression Scale

As you are pregnant or have recently had a baby, we would like to know how you are feeling. Please check the answer that comes closest to how you have felt IN THE PAST 7 DAYS, not just how you feel today.

In the past 7 days:

For peer review only

1. I have been able to laugh and see the funny side of things
    a. As much as I always could
    b. Not quite so much now
    c. Definitely not so much now
    d. Not at all
2. I have looked forward with enjoyment to things
    a. As much as I ever did
    b. Rather less than I used to
    c. Definitely less than I used to
    d. Hardly at all
3. I have blamed myself unnecessarily when things went wrong
    a. Yes, most of the time
    b. Yes, some of the time
    c. Not very often
    d. No, never
4. I have been anxious or worried for no good reason
    a. No, not at all
    b. Hardly ever
    c. Yes, sometimes
    d. Yes, very often
5. I have felt scared or panicky for no very good reason
    a. Yes, quite a lot
    b. Yes, sometimes
    c. No, not much
    d. No, not at all
6. Things have been getting on top of me
    a. Yes, most of the time I haven't been able to cope at all
    b. Yes, sometimes I haven't been coping as well as usual
    c. No, most of the time I have coped quite well
    d. No, I have been coping as well as ever
7. I have been so unhappy that I have had difficulty sleeping
    a. Yes, most of the time
    b. Yes, sometimes
    c. Not very often
    d. No, not at all
8. I have felt sad or miserable
    a. Yes, most of the time
    b. Yes, quite often
    c. Not very often
    d. No, not at all
9. I have been so unhappy that I have been crying
    a. Yes, most of the time
    b. Yes, quite often
    c. Only occasionally
    d. No, never
10. The thought of harming myself has occurred to me
    a. Yes, quite often
    b. Sometimes
    c. Hardly ever
    d. Never

For peer review only

For peer review only

BRIGHTSELF

Welcome Guide (Arm 2)

Version1.0 - 6 Jul 2016

Welcome

Hello, and thank you for collaborating with this study to test the feasibility of BrightSelf, an application for the assessment of psychological wellbeing during pregnancy.

This application and study are the result of a collaboration between Imperial College London and Trinity College Dublin.

Here you will find a quick explanation of BrightSelf, the value it provides to participants, and guidelines for recruitment.

The following section answers any questions the user may have and might be of use to you during recruitment.

For peer review only

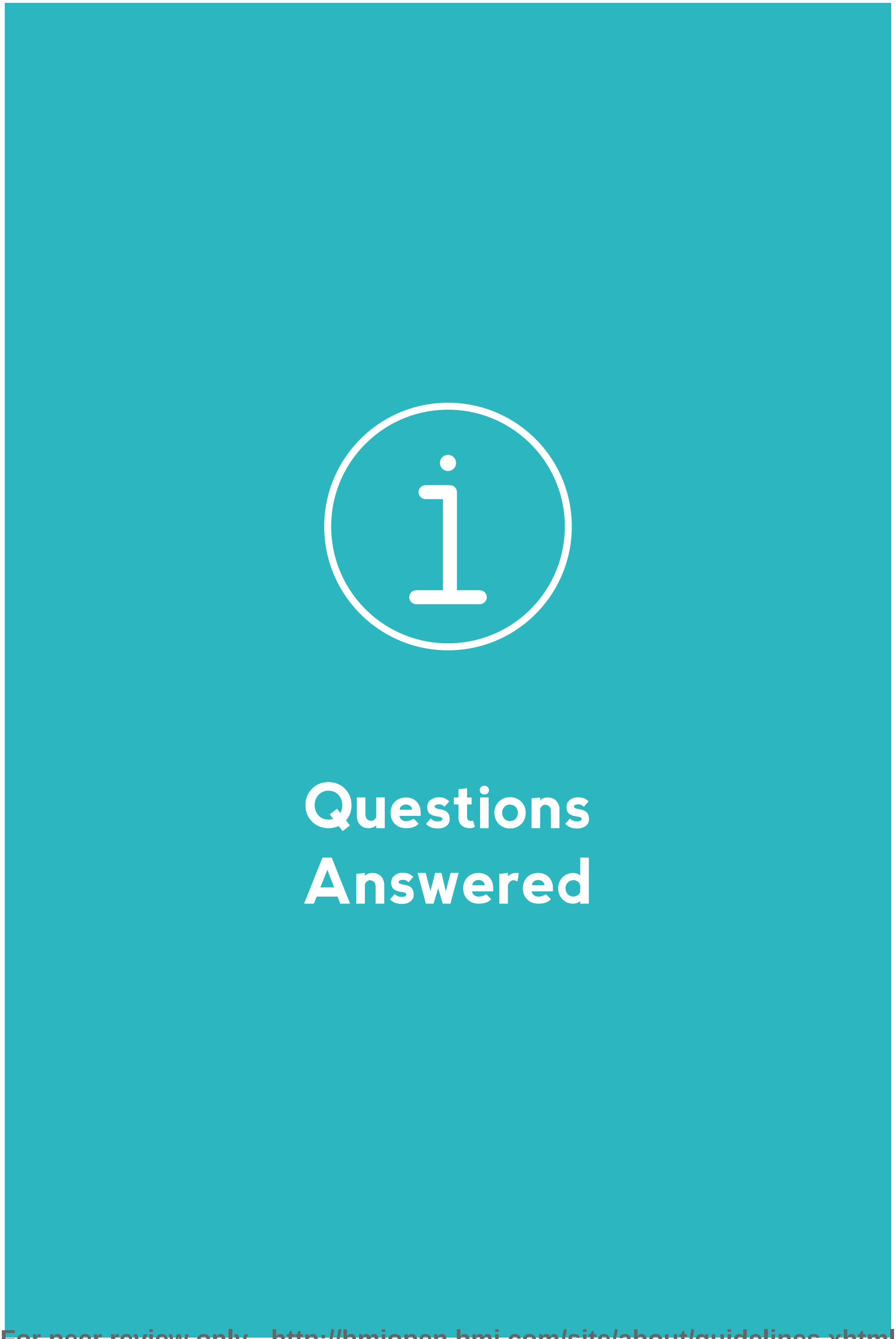

For peer review only - http://bmjopen.bmj.com/site/about/guidelines.xhtml

What is BrightSelf?

BrightSelf is a new mobile app designed to help women develop a picture of their psychological wellbeing during pregnancy.

Researchers at Imperial College London and Trinity College Dublin are currently assessing the engagement of mothers-to-be with BrightSelf. In future, we hope to assess the value of the app to improve the wellbeing of mothers-to-be and communication between women and their healthcare providers.

By **checking in** you can capture your mood, sleep, energy and more in the moment. By **checking back**, BrightSelf allows you to build a picture of your wellbeing over the past week. BrightSelf also provides the ability to interact with your data, information on wellbeing and research, sources of professional support, and an ideas machine, your personal source for a pick-me-up!

For peer review only - http://bmjopen.bmj.com/site/about/guidelines.xhtml

What is expected of users?

BrightSelf is here for you to use in any way you find useful. You can make self reports according to a schedule you determine for yourself or simply as often as you feel useful. We have tried to design BrightSelf so that it is quick and easy to use and ultimately supports your needs.

As part of our research to examine the value of BrightSelf and technologies for the report of psychological wellbeing, you will be prompted infrequently over the course of 6 months to check and and check back using the application.

These prompts will appear as notifications on the front screen of your phone or as a banner appearing at the top.

For peer review only - http://bmjopen.bmj.com/site/about/guidelines.xhtml

For peer review only

These reports will be used entirely anonymously to inform our research and we would greatly appreciate if you could respond to the requests as accurately and timely as possible. At the end of this period you may then also be asked to complete a short questionnaire detailing your thoughts on the experience.

Through your feedback, and by examining, anonymously and securely, these responses, we hope to move towards improving the experience of all around the time of pregnancy.

The next section provides a quick guide to the installation and use of BrightSelf.

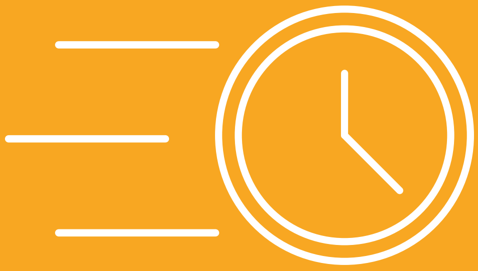

A Quick Guide

For peer review only - http://bmjopen.bmj.com/site/about/guidelines.xhtml

The Android Play Store Icon

The Apple App Store Icon

To use BrightSelf, the first step is to download the app to a phone by visiting the appropriate **app store**.

For peer review only

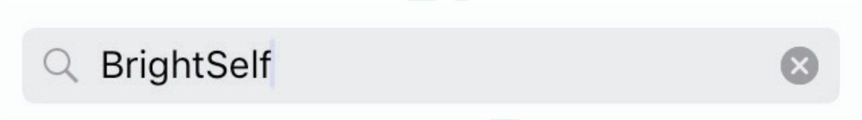

You can then search the store for BrightSelf. Look for the app icon and when you find it, choose to **install** it. It’s free!

For peer review only

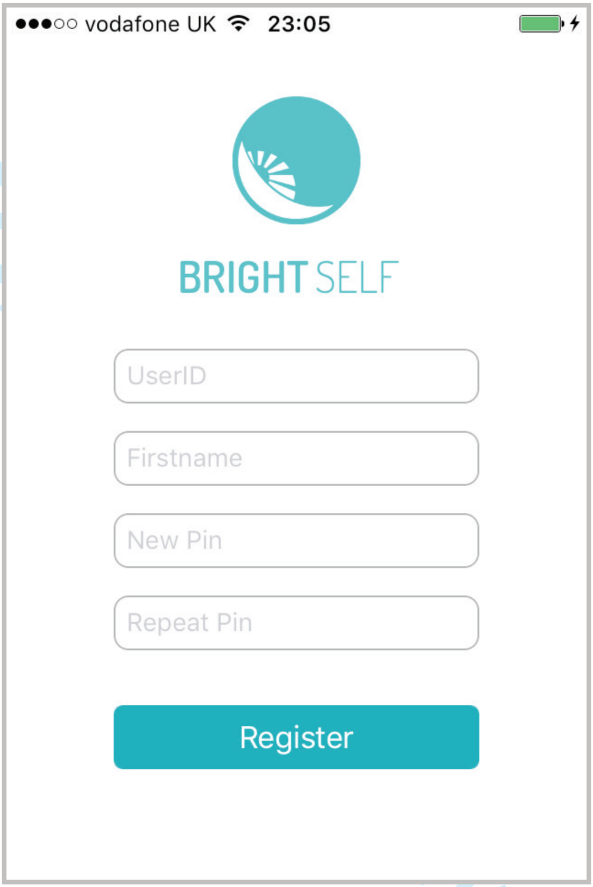

When you first open BrightSelf you will need to **register** a new user.

You may first be asked whether you wish to receive **push notifications** from BrightSelf.

If asked, please accept to do so!

In order to register, an internet connection (Wifi or mobile internet) is required. Once the device is connected, the user simply needs to enter the following information;

- **A 9-digit UserID** provided by the researchers. Please enter this carefully.
- The user's **Firstname** only (without numbers or spaces)
- **A 4-digit PIN** of the user's choosing which will allow her to login in the future. Please ensure to pick a pin which will not be forgotten!
- Enter this **PIN** a second time to ensure a match.

On clicking register, the system will then register the user for the study in several seconds. That's it, you're ready to explore BrightSelf!

For peer review only - http://bmjopen.bmj.com/site/about/guidelines.xhtml

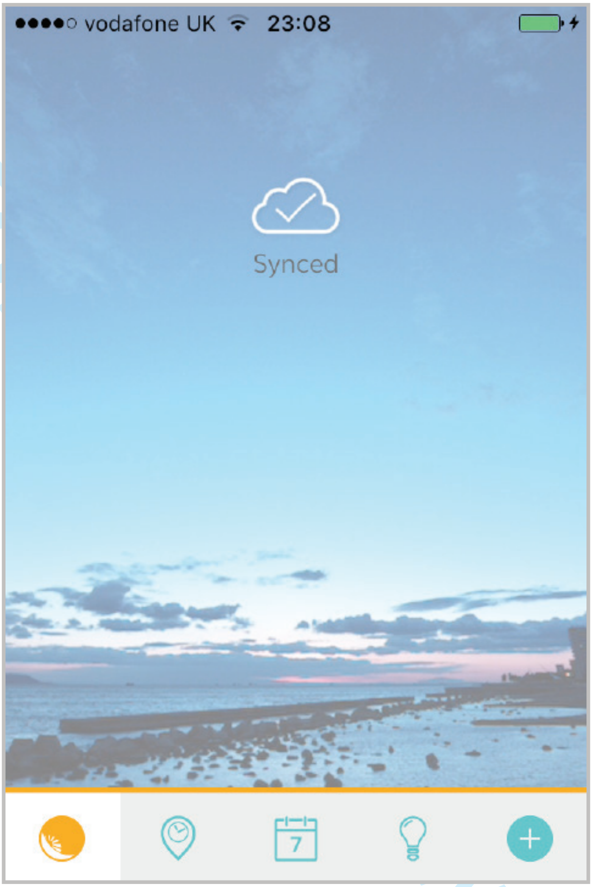

On the **home screen**, the syncing button shows whether your most recent reports have been uploaded, anonymously and securely, to our server for inclusion in our research. You can navigate through the app using the navigation bar located at the bottom of the screen.

For peer review only - http://bmjopen.bmj.com/site/about/guidelines.xhtml

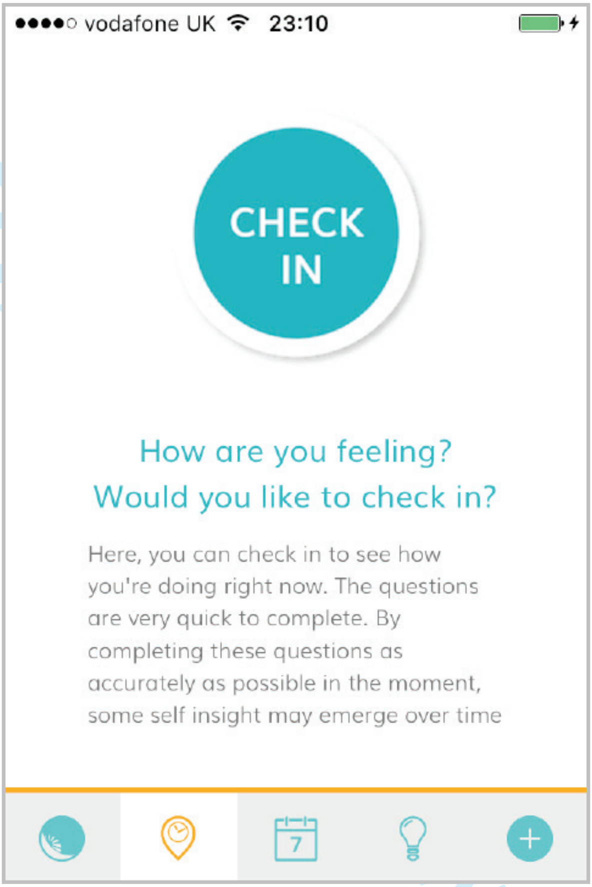

Checking in allows you to note how you are feeling right now by very quickly rating your mood, sleep, worry, enjoyment and energy.

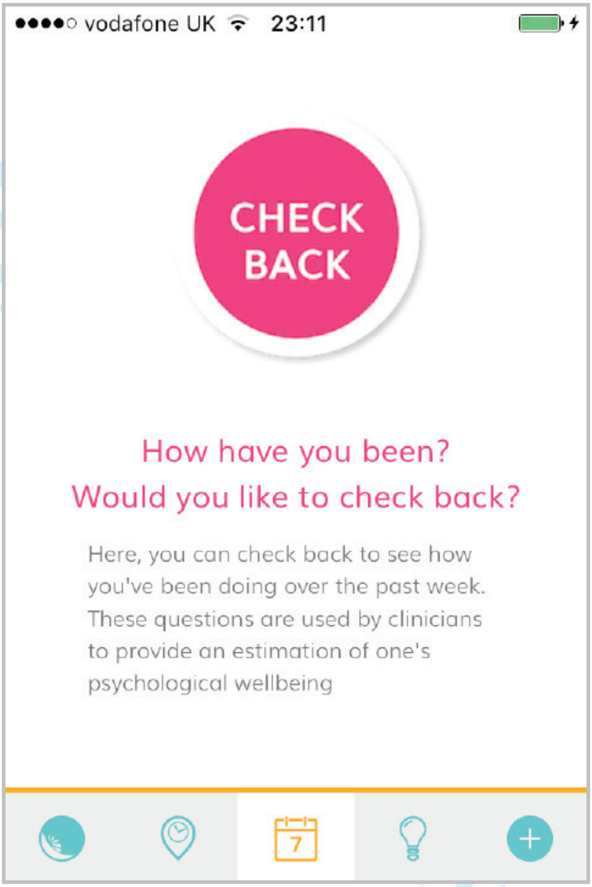

Checking back allows you to make note of your wellbeing over the past week by answering 10 questions which are normally used by clinicians to assess mood during pregnancy.

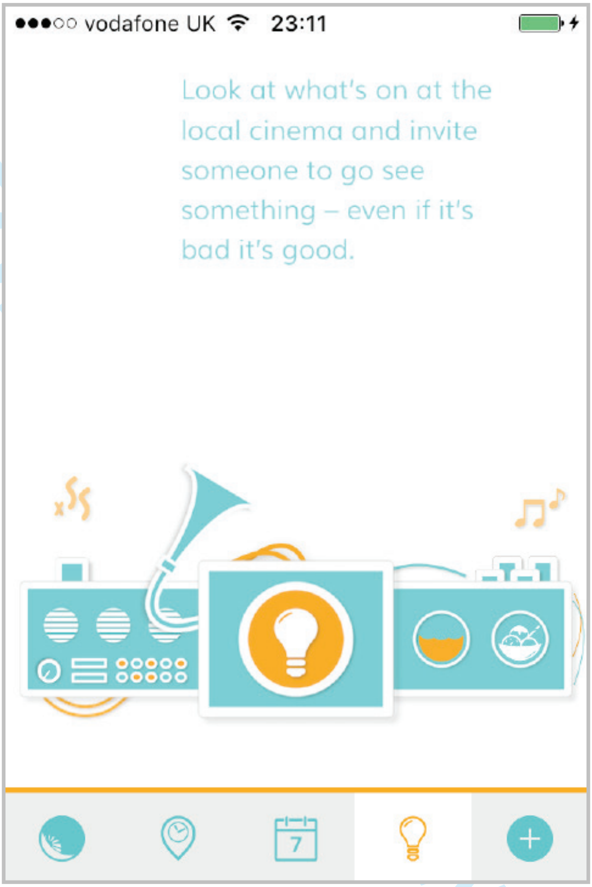

The **ideas machine** is there to provide that pick-me-up we all need from time to time. Remember however, that BrightSelf is not intended as a replacement for professional help. If at any point you feel that you need extra support, please do contact your GP or midwife.

For peer review only - http://bmjopen.bmj.com/site/about/guidelines.xhtml

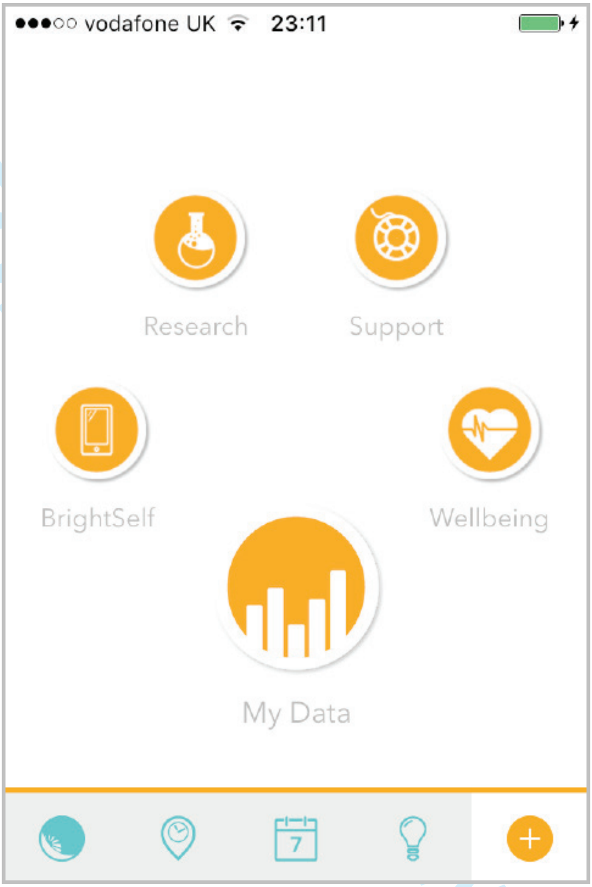

The **extras** section contains information about BrightSelf, wellbeing, and our research. Here you will also find additional sources of professional support should you need them.

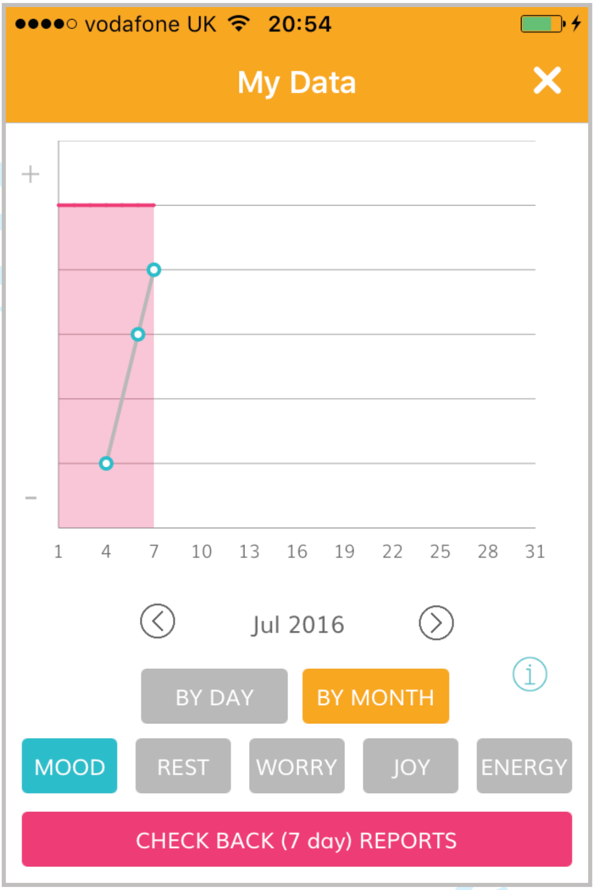

Under **My Data** you can interact with a plot of all your previous responses, allowing you to examine your reports over time, and empowering you to take control of your own wellbeing. Please note however, that these graphs do not represent any proven clinical value.

Finally, please note that you will receive infrequent **notifications** over the next 6 months which will request you to check in and check back. How these notifications appear will depend upon your device and some examples are shown below.

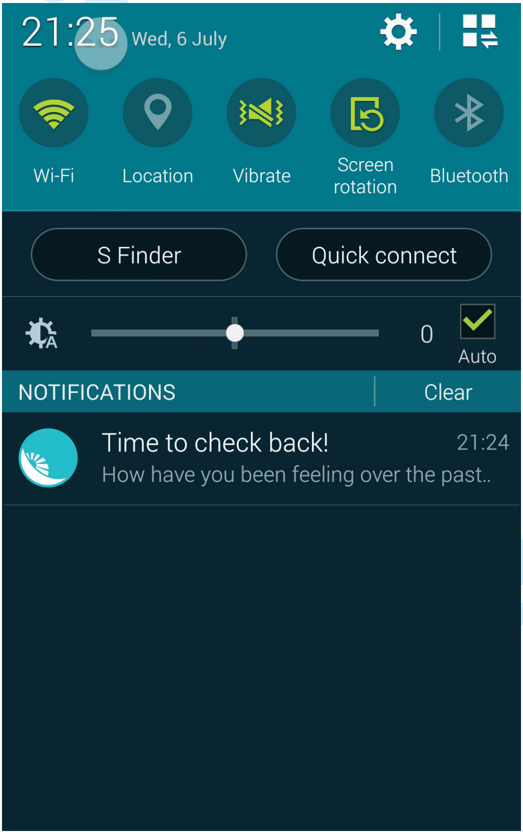

Android Notification

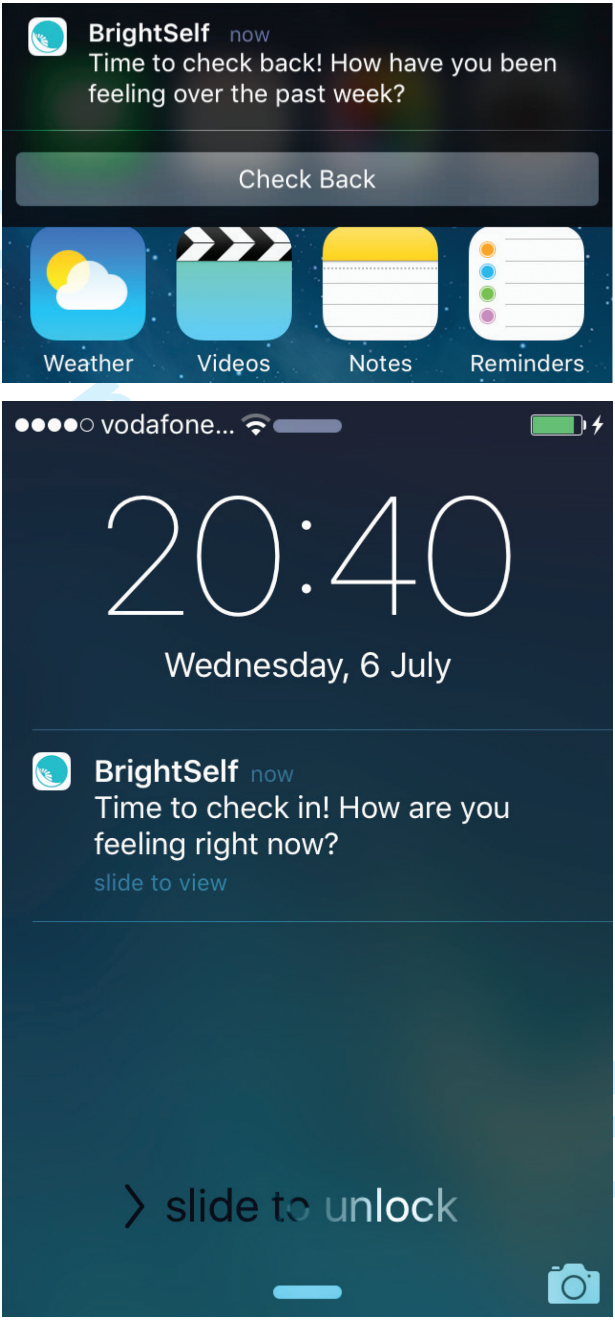

Apple Notification

For peer review only - http://bmjopen.bmj.com/site/about/guidelines.xhtml

Administrative Notes

Please note that the user's data is captured anonymously throughout the duration of the study and transferred to our servers using a secure connection.

No-one outside the research team will have access to the user's data. Any data gathered will be used for research purposes only.

This app is not intended to substitute the woman's care in any way but only to complement it. Please inform the user that if at any point she feels the need for extra support to contact her GP or midwife.

If any user's responses do indicate that she is struggling and might benefit from extra support, the system will alert the research team who will then contact the local research team to follow up.

For peer review only - http://bmjopen.bmj.com/site/about/guidelines.xhtml

Contact

At all times, our primary concern is with the user's wellbeing, privacy, and security.

Should you or any user have questions at any time please don't hesitate to contact us at **help@brightselfresearch.org**

Thank you once again for your participation and we hope you find participating in BrightSelf a valuable experience!

For peer review only

For peer review only

Appendix 3. Momentary and contextual questions

Momentary questions

1. I feel…
    a. Great
    b. Good
    c. Ok
    d. Not good
    e. Not good at all
2. How rested do I feel?
    a. Very rested
    b. Somewhat rested
    c. Neither rested nor tired
    d. Somewhat tired
    e. Very tired
3. How worried do I feel?
    a. Very worried
    b. Quite worried
    c. Somewhat worried
    d. A little worried
    e. Not at all worried
4. Am I enjoying myself?
    a. A lot
    b. Quite a lot
    c. Somewhat
    d. Not much
    e. Not at all
5. How energetic do I feel?
    a. Very energetic
    b. Fairly energetic
    c. Moderately energetic
    d. Not very energetic
    e. Not at all energetic

Contextual questions

1. Right now, I am…
    a. At home
    b. At work
    c. At the shops
    d. Outside
    e. At a Café or Restaurant
    f. In Transport
    g. At a Friend's or Family
    h. In a Clinic
    i. Other
2. Right now, I am…
    a. Relaxing

For peer review only

b. Working
c. Shopping
d. Looking After Others
e. Eating or Drinking
f. Travelling
g. Exercising
h. Waiting
i. None

For peer review only

Appendix 4. Post-study acceptance survey

Please rate your agreement with the following statements with respect to this system:

1. This app is easy to use
   - Strongly agree – Strongly disagree (7-point Likert Scale)
2. I learned to use this app quickly
   - Strongly agree – Strongly disagree (7-point Likert Scale)
3. I would recommend this app to a friend
   - Strongly agree – Strongly disagree (7-point Likert Scale)
4. I would repeat the experience of using this app
   - Strongly agree – Strongly disagree (7-point Likert Scale)
5. I found using this app an engaging experience
   - Strongly agree – Strongly disagree (7-point Likert Scale)
6. I found the assessments useful
   - Strongly agree – Strongly disagree (7-point Likert Scale)
7. I was asked to provide reports:
   - Much too infrequently
   - Too infrequently
   - The right amount
   - Too frequently
   - Much too frequently
8. The experience of using this app met my needs:
   - Strongly agree – Strongly disagree (7-point Likert Scale)
9. What were your motivations in using this app?
10. What did you like the most about the experience of using this app?
11. What did you like least about the experience of using this app?
12. How would you improve the experience of using this app?
13. Do you have any other comments that you would like to make?

To be answered by those participants allocated to the retrospective plus momentary assessment strategy:

14. I found it useful to compare reports made right now with those made over the past 7 days:
    - Strongly agree – Strongly disagree (7-point Likert Scale)

For peer review only
